# ALKBH4 impedes 5-FU Sensitivity through suppressing GSDME induced pyroptosis in gastric cancer

**DOI:** 10.1038/s41419-024-06832-1

**Published:** 2024-06-20

**Authors:** Xin Jiang, Zhiman Zhu, Lina Ding, Wenqi Du, Dongsheng Pei

**Affiliations:** 1 Department of Pathology, School of Basic Medical Sciences, Xuzhou Medical University, Xuzhou, 221004 China; 2 Department of Human Anatomy, School of Basic Medical Sciences, Xuzhou Medical University, Xuzhou, 221004 China

**Keywords:** Cancer, Biomarkers

## Abstract

5-Fluorouracil (5-FU) is the primary treatment option for advanced gastric cancer. However, the current challenge lies in the absence of validated biomarkers to accurately predict the efficacy and sensitivity of 5-FU in individual patients. It has been confirmed that 5-FU can regulate tumor progression by promoting gasdermin E (GSDME, encoded by DFNA5) cleavage to induce pyroptosis. Lysine demethylase ALKBH4 has been shown to be upregulated in a variety of tumors to promote tumor progression. However, its role in gastric cancer is not clear. In this study, we observed a significant upregulation of ALKBH4 expression in gastric cancer tissues compared to adjacent normal tissues, indicating its potential as a predictor for the poor prognosis of gastric cancer patients. On the contrary, GSDME exhibits low expression levels in gastric cancer and demonstrates a negative correlation with poor prognosis among patients diagnosed with gastric cancer. In addition, we also found that high expression of ALKBH4 can inhibit pyroptosis and promote the proliferation of gastric cancer cells. Mechanistically, ALKBH4 inhibits GSDME activation at the transcriptional level by inhibiting H3K4me3 histone modification in the GSDME promoter region, thereby reducing the sensitivity of gastric cancer cells to 5-FU treatment. These findings provide further insight into the regulatory mechanisms of ALKBH4 in the progression of gastric cancer and underscore its potential as a prognostic marker for predicting the sensitivity of gastric cancer cells to 5-FU treatment.

## Introduction

Gastric cancer is one of the leading causes of cancer-related deaths worldwide [1]. Despite advancements in diagnosis and treatment, the overall survival rate for gastric cancer remains low [2]. At present, chemotherapy is still one of the standard therapies for gastric cancer [3]. 5-FU is a well-established and widely used chemotherapeutic agent for the treatment of gastrointestinal and other solid tumors. Nevertheless, the precise mechanism underlying the development of resistance to 5-FU-based chemotherapy remains incompletely understood, necessitating further investigation [4]. Hence, there is an immediate need to identify novel therapeutic targets and develop strategies aimed at enhancing patients’ sensitivity to chemotherapy, ultimately leading to improved prognosis [5].

Pyroptosis is a form of cell death characterized by the activation of the Caspases family through inflammasomes, leading to the cleavage of gasdermin family members and the subsequent secretion of pro-inflammatory cytokines [6, 7]. Importantly, the emergence of pyroptosis provides a new opportunity for chemotherapy-insensitive tumors [8]. Studies have demonstrated that pyroptosis serves as a regulator of the therapeutic response of tumors, and enhancing drug-induced pyroptosis can overcome drug resistance in tumors [8, 9]. The mechanism of pyroptosis can be categorized into two pathways: classical pathway and non-classical pathway. In the classical pathway, activation of inflammasomes leads to the activation and cleavage of Pro-Caspase 1, resulting in the generation of active Caspase 1 [10]. On the one hand, activated Caspase 1 cleaves GSDMD to produce N-terminal fragment N-GSDMD, which then penetrates the cell membrane to form pores. On the other hand, IL-1β and IL-18 precursors are cleaved by activated Caspase 3 to produce mature IL-1β and IL-18, thereby amplifying the inflammatory response [11, 12]. In the non-classical pathway, Caspase 4, Caspase 5, and Caspase 11 directly bind to lipopolysaccharide (LPS), initiating the non-classical pyroptosis process [13]. This process leads to cell membrane perforation, cell lysis, and inflammatory response [14]. Emerging research findings have demonstrated that chemotherapy can trigger the activation of Caspase 3, leading to the cleavage of GSDME and the subsequent generation of N-GSDME, ultimately culminating in pyroptosis of cancer cells. Multiple research studies have suggested that GSDME has the potential to serve as an important target for tumor chemotherapy [15–18].

The family of AlkB homolog (ALKBH) proteins, which are homologs of the Escherichia coli AlkB dioxygenase and contain a 2-oxoglutarate (2OG) binding site, play an essential role in a number of important regulatory processes in eukaryotic cells, including the repair of alkylation lesions in DNA, RNA, and nucleoprotein complexes [19]. A total of nine humans and thirteen Arabidopsis thaliana ALKBH proteins have been identified, which exhibit a range of diverse functions [19]. Recent studies have demonstrated that ALKBH1 [20], ALKBH5 [21–24], ALKBH8 [19], and FTO [25, 26] function as tRNA demethylases, which are associated with a range of diseases, including obesity, osteoporosis, diabetes and multiple cancers [27, 28]. In humans, FTO regulates adipogenesis through tyrosine protein kinase, Cyclin-A2, and cyclin-dependent kinase 2, demonstrating an interaction between FTO and obesity at multiple levels of human metabolism [19, 29]. And in mice, perturbation of FTO expression affects body weight. Lack of FTO protein leads to growth retardation, reduced body weight, white adipose tissue, and increased energy metabolism, while overexpression of FTO leads to obesity in mice [30–32]. However, in humans, mutations in the FTO gene lead to more severe phenotypic changes. Studies have shown that mutations in the FTO gene mutations lead to structural brain malformations, heart defects, and genital defects in humans, and in general, rodents and humans exhibit differences in pathophysiology, suggesting that the FTO gene plays a more complex role in humans than it does in rodents [19, 33, 34]. ALKBH2 and ALKBH3 are DNA demethylases that may serve as cancer indicators [35–39]. ALKBH4 may be a new cell division factor. ALKBH4 was discovered to be a K84mel actin demethylase that modulates the actin-myosin II interaction. The involvement in tumourigenesis is not fully clarified [40]. Few studies have concentrated on the molecular structure or functions of ALKBH6, which remain to be determined in the future [19, 41]. However, it has been reported that ALKBH6 may be involved in rhabdomyosarcoma development [41], breast cancer progression [42], and pancreatic cancer cell survival [43]. Among all homologs of the EcAlkB protein, only ALKBH7 contains conserved mitochondrial signals that are localized in the mitochondrial matrix and are involved in fatty acid metabolism in mice and alkylation and oxidation-induced programmed necrosis in humans. In our subsequent research, we observed a significant upregulation of demethylase ALKBH4 in gastric cancer. Nevertheless, the impact of altered ALKBH4 expression in gastric cancer cells on disease progression and chemotherapy sensitivity remains unclear. In this study, we found that ALKBH4 inhibited the expression of GSDME in gastric cancer, thereby reducing 5-FU-induced pyroptosis, eventually leading to reduced sensitivity of gastric cancer cells to 5-FU treatment and promoting gastric cancer progression.

## Materials and methods

### Clinical specimens

Clinical specimens were obtained from the Affiliated Hospital of Xuzhou Medical University, China. The patients had not received radiotherapy or chemotherapy prior to surgery. The study was conducted in accordance with the guidelines of the Declaration of Helsinki. The study design, study protocol, and information safety were approved by the Ethics Committee of the Affiliated Hospital of Xuzhou Medical University, and written informed consent was obtained from all patients before participation in the study program. Case inclusion criteria: the patient was first diagnosed with gastric cancer; the patient’s cardiopulmonary function was normal, the electrocardiogram result as well as the cardiac function test were within the normal range, and there was no history of serious cardiopulmonary disease in the past; liver and kidney function were normal; blood system examination was normal; the patient’s basic personal information and clinical data were complete, and there was no complication of other malignant tumors; case exclusion criteria: the patient’s data had omissions; there was a history of other malignant tumors before the diagnosis was confirmed; the patient was unable to receive relevant examination or treatment due to his physical status; the mental status was poor after the operation, and the prognosis was affected due to his own poor underlying physical conditions.

### Cell culture

Human gastric cancer cell lines (MKN45, HGC-27, AGS, MGC803) and Human Gastric Epithelial Cells (GES-1) were derived from the Shanghai Institute of Cell Research, Chinese Academy of Sciences (Shanghai, China). All cells were identified by STR (short tandem repeat) and tested for mycoplasma contamination. AGS cells originated from human gastric adenocarcinoma. MGC803 cells originated from human gastric hypo-differentiated mucinous-like adenocarcinoma. MKN45 cells originated from liver metastases of human poorly differentiated gastric adenocarcinoma. HGC-27 cells originated from human undifferentiated gastric cancer. GES-1 cell line originated from the fetal gastric mucosal epithelium. Human gastric fibroblasts originated from fibroblast-like cells of surgically resected normal gastric tissue. MKN45 cell line was grown in Dulbecco’s modified Eagle’s medium (DMEM; Bio-Channel Biotechnology, Nanjing, China) supplemented with 10% (vol/vol) fetal bovine serum (FBS; Gibco, Grand Island, NY, USA) and 2 mM L-glutamine. RPMI-1640 (Bio-Channel Biotechnology, Nanjing, China) was used to cultivate the HGC-27 cell line. Both of them were incubated with penicillin-streptomycin (100 U/ml, Gibco, USA) at 37 °C in a humidified environment containing 5% CO2.

### Gene transfection and knockdown

ALKBH4 plasmid was synthesized and cloned into pcDNA3.1 vector (shhebio, Shanghai, China). The plasmids were transfected into MKN45 and HGC-27 cells using lipofectamine 2000 (Invitrogen, USA) according to the instructions. In order to obtain a stable cell line, GC cells were infected with lentiviral vector plasmid (obiosh, Shanghai, China) shALKBH4, and short hairpin RNA (shRNA) lentivirus.

### Western blot analysis

Cells were lysed with RIPA lysis buffer (Beyotime, Shanghai, China) supplemented with protease inhibitor phenylmethanesulfonyl fluoride (Vicmed, Xuzhou, China). Cell samples were collected using a scraper and centrifuged at 14,000 rpm at 4 °C for 15 min to prepare a whole cell lysate. Protein concentration was determined using the bicincinho acid protein assay kit (KeyGEN, Jiangsu, China). After electrophoresis, the protein on the SDS-PAGE gel was transferred to the nitrocellulose membrane. After blocking with 5% skim milk for 2 h, the primary antibody was incubated at 4 °C, and the secondary antibody coupled with horseradish peroxidase (HRP) was incubated the next day. Finally, the chemiluminescence image analysis system (Tanon, Shanghai, China) was used for imaging. The primary antibodies used for western blotting are as follows: anti-ALKBH1 (A14079, ABclonal), anti-ALKBH2 (821447, Zenbio), anti-ALKBH3 (A5808, ABclonal), anti-ALKBH4 (A12593, ABclonal), anti-ALKBH5 (A11684, ABclonal), anti-ALKBH6 (M30162, ABmart), anti-ALKBH7 (A2331, ABclonal), anti-ALKBH8 (A7142, ABclonal), anti-FTO (A1438, ABclonal), anti-GSDME (ab215179, Abcam), anti-GSDMD (A20728, ABclonal), anti-H3K4me3 (A22146, ABclonal), anti-H3K4me1 (A22078, ABclonal), anti-H3K9me3 (A2360, ABclonal), anti-H3K36me3 (A20379, ABclonal), anti-H3K56me3 (A7260, ABclonal), anti-H3K64me3 (A7259, ABclonal), anti-Caspase 1 (A0964, ABclonal), anti-Caspase 3 (A16793, ABclonal), anti-β-actin (AC026, ABclonal), Histione H3 (ab1791, Abcam).

### RNA isolation and real-time quantitative reverse transcription PCR (qRT-PCR)

TRIzol Reagent (Vazyme, Nanjing, China) was used to isolate total RNA from gastric cancer tissues and cells according to the manufacturer’s protocol. A microgram of RNA was reversely transcribed into complementary DNA (cDNA) for qPCR using the Prime Script RT reagent Kit (Servicebio, Beijing, China). The mRNA level was assessed by SYBR Premix Ex Taq (Servicebio, Beijing, China). The relative expression of mRNA was quantified by comparing the cycle threshold method (2-ΔΔCt). Primer sequences are listed in Table 1.

**Table 1:** List of PCR primers used in the study.

Primers	Primer sequences (5′ to 3′)
ALKBH4-Forward primers	CACCTGGAATGTTTCAAATGC
ALKBH4-Reverse primers	TCCTGTTCGCACACTATGTCA
GAPDH-Forward primers	GAAGGTGAAGGTCGGAGTC
GAPDH-Reverse primers	GAAGATGGTGATGGGATTTC
GSDME-Forward primers	CCAGTTTTTATCCCTCACCCTTG
GSDME-Reverse primers	CAAACTTGCCCTCGTATTTCACA
GSDMD-Forward primers	GCTGGTTATTGACTCTGACTTGGAC
GSDMD-Reverse primers	GACCCCATCTGTCAGGAAGTT
NLRP3-Forward primers	CTGGCATCTGGGGAAACCT
NLRP3-Reverse primers	TCTCTCCTGTTGATCGCAGC
Caspase 1-Forward primers	ACTGAGGCCGTGTGCAATTA
Caspase 1-Reverse primers	CAACACAGCAACCAGCAGAC

### Immunohistochemistry (IHC)

For IHC staining, we collected 87 cases of normal gastric tissues and 93 cases of gastric cancer tissues from the Affiliated Hospital of Xuzhou Medical University. The use of human gastric cancer tissue specimens was approved by the Ethics Committee of the First Affiliated Hospital of Xuzhou Medical University. The tissue processing involved fixing the tissue in the Leica TP1020 tissue processor, manufactured by Leica Biosystems in Germany. According to the manufacturer’s instructions, IHC staining was performed using the Vectastain ABC kit (Vector Laboratories). Paraffin-embedded human gastric cancer tissue sections were stained with ALKBH4 and GSDME antibodies. In addition, IHC staining was performed on paraffin-embedded xenograft sections using ALKBH4 (A12593, ABclonal), GSDME (ab215179, Abcam), and Ki67 (A25399, ABclonal) antibodies.

### Evaluation of immunostaining

For IHC scoring method: staining intensity score multiplied by the percentage of positive cells. The staining intensity: 0 (negative), 1 (weak positive), 2 (moderate), 3 (strong positive); for the percentage of positive cells: 1 point (0~25% of tumor cells stained), 2 points (25%~50% of tumor cells stained), 3 points (51%~75% of tumor cells stained), 4 points (76%~75% of tumor cells stained).

### Assessment of in vivo tumor growth

BALB/c mice aged 4–6 weeks were procured from the Animal Research Center of Xuzhou Medical University under SPF standard conditions. The pre-experimental results indicated that 36 nude mice were purchased. Mice were randomized into different groups and analysis was blinded. Random allocation of subjects was achieved using Excel [1]: 36 mice were numbered from 1 to 36 according to weight [2]. Add a column of random numbers labeled Random1, input “=RAND ()” to generate random numbers, and sort Random1 into categories: shNC + vehicle, shALKBH4 + vehicle, shNC + 5-FU, shALKBH4 + 5-FU, shALKBH4 + shGSDME, and shALKBH4 + shGSDME + 5-FU. Lentivirus-transfected cells were subsequently implanted into the dorsal region of each mouse at a density of 2 × 10^7^ cells/mouse. Once the tumor volume had reached a size of 20–50 mm³, 5-FU (20 mg/kg) was administered intraperitoneally to the spiked group, while the control solvent was injected intraperitoneally to the non-spiked group. The tumor size was measured using a caliper, and tumor volume was calculated using the formula (length × width × width)/2. Following a 5-week injection period, the mice were euthanized, and the tumors were collected, photographed, and weighed. The animal experiments involved in this study were approved by the Animal Ethics Committee of Xuzhou Medical University (approval number: 202210S040).

### Cell viability assay

GC cells were pre-transfected with plasmids overexpressing ALKBH4 and control vectors, and MKN45 and HGC-27 cells were inoculated in a 96-well plate at a concentration of 5000 cells per well in 200 μL medium. After culture to the specified time, the medium was removed and 100 μL of diluted CCK8 solution (APExBIO, USA) was added. The plate was incubated at 37 °C for 2 h, and the absorbance was measured at 450 nm. All experiments were conducted in three independent experiments.

### In vitro assays of drug sensitivity

The treated cells were cultured in 100 μL culture medium at a concentration of 5000 cells per well and placed overnight. The medium was then sucked out and supplemented with a medium containing the required concentration of 5-Fluorouracil. After incubation to the specified time, the medium was removed, and 100 μL of diluted CCK8 solution (APExBIO, USA) was added and incubated at 37 °C for 2 h. The absorbance was measured at 450 nm. The formula for calculating cell viability is as follows: cell viability rate = [(As − Ab)/(Ac − Ab)] × 100, where As represents the absorbance of the experimental well, Ab represents the absorbance of the blank well, and Ac represents the absorbance of the blank control well.

### Cell proliferation curves were plotted and IC50 values were calculated

Following the input of the converted absorbance values into GraphPad Prism 8.2.1 software, the axes were constructed with the inhibition rate and concentration. Thereafter, logarithmic transformation was performed to calculate the IC50 of 5-FU for the gastric cancer cell lines HGC-27 and MKN45. This was achieved by selecting “ Log (inhibitor) vs. normalized response—Variable slope “ under the option of “Dose-Response-Inhibition” in the nonlinear regression (curve fitting).

### Colony formation assays

The pretreated cells were seeded in 6-well plates at a concentration of 1000 cells per well. Cells were cultured in serum-free DMEM, and the cell culture medium was changed every 3 days. After 2 weeks of culture, the culture medium was removed, and the colonies were fixed in 10% formalin solution for 2 h, and then stained with 0.1% crystal violet solution at room temperature for 15 min. Colony counting was performed using Image J software (version 1.8.0), and the digital image of the plate was collected as a permanent record.

### PI staining

PI staining kit (Yeasen, Shanghai, China) was used to evaluate pyroptosis cell death. The treated gastric cancer cells were collected and centrifuged at 4 °C, 1000 × *g* for 3–5 min to precipitate the cells to the bottom of the tube. Carefully remove the supernatant, take the above collected 10^5^–10^6^ cells were resuspended in 0.8–1 mL cell staining buffer and photographed using an inverted fluorescence microscope (Olympus, Tokyo, Japan).

### TUNEL assay

Cells were divided into shNC, shALKBH4, shALKBH4 + 5-FU, shALKBH4 + 5-FU + DMF and positive control groups. The pretreated cell slides were fixed in 4% paraformaldehyde. TUNEL detection was then performed according to the manufacturer’s instructions (Beyotime Biotechnology, China). Finally, the slides were visualized by fluorescence microscope.

### Trypan blue staining assay

After different treatments, GC cells were collected, prepared, and stained with 0.4% trypan blue reaction buffer (Sigma–Aldrich, USA) at room temperature for 20 min. After that, the cells were observed under an optical microscope, and the dead blue cells were counted. Finally, the cell survival rate was calculated by the following formula: cell death rate (%) = 1- (total cells-dead blue cells)/total cells × 100%.

### AnnexinV/7- AAD (amino-actinomycin D) staining

The pretreated gastric cancer cells were seeded in 6-well plates at a density of 0.2 × 10^6^/well. Subsequently, the cells were exposed to 5-FU for a duration of 24 h. Following the treatment period, both adherent and exfoliated cells were harvested and rinsed with pre-cooled PBS. The cells were then resuspended in 100 μL of 1× binding buffer and subjected to staining with 5 μL of annexin v-FITC and 10 μL of 7-AAD (Yeasen) in darkness at room temperature for 15 min. Subsequently, 400 μl of binding buffer was added, and the cells were analyzed using flow cytometry.

### LDH release assay

Cells were cultured in 96-well plates and then subjected to different treatments. One hour before the assay, 20 μL of LDH release agent was added to the culture medium. LDH release levels were assessed by using LDH Cytotoxicity Assay Kit II (Beyotime, Shanghai, China), according to the manufacturer’s instructions. Cytotoxicity or mortality (%) = (Absorbance of sample - Absorbance of sample control wells) × (Absorbance of maximum cellular enzyme activity - Absorbance of sample control wells) × 1/2 × 100.

### Microscopic imaging

In order to observe the morphology of pyroptosis cells, the pretreated cells were seeded in 6-well plates at a density of about 60%. Olympus IX71 microscope (Olympus Co, Tokyo, Japan) was used to capture images of bright-field cells after treatment with 5-FU.

### Transmission electron microscope (TEM)

First, we treated and amplified MKN45 and HGC-27 cell lines to collect cells at the logarithmic growth phase and fixed them to maintain their morphological structure. Then, dehydration treatment was carried out, and organic solvents were used to gradually replace water. Next, the cell samples were immersed in a suitable resin for impregnation and embedding. Subsequently, the solidified sample is cut into extremely thin slices and transferred to the copper grid. Finally, TEM was used to observe and photograph the pores generated during pyroptosis.

### Chromatin immunoprecipitation assay (ChIP)

For four groups of cells (OEALKBH4, Vector, shALKBH4, shNC), the stock solution was discarded and replaced with 10 ml of fresh complete medium, then 280 μl of 37% formaldehyde was added and incubated for 10 min at room temperature. To stop the reaction, 1 M glycine was added until a final concentration of 0.125 M was reached. Following the manufacturer’s protocol, ChIP analysis was performed using high-sensitivity kits (#ab185913, Abcam) and anti-ALKBH4 (A12593, abcam) antibody, anti-H3K4me3 (A22146, ABclonal) antibody. For RT-qPCR analysis of the input and output DNA, TB Green Advantage qPCR Premix (#639676, Takara) was utilized.

### Differential expression analysis

In the analysis of TCGA tumors, the DiffExp module facilitates the investigation of differential gene expression between tumors and adjacent normal tissues. A box plot was utilized to visually represent the distribution of ALKBH4 expression levels, while the statistical significance of differential expression was assessed using the Wilcoxon test. In the case of normal data available, these genes are presented in gray-shaded columns.

### Receiver operating characteristic (ROC) curve

ROC curve is a commonly used method to evaluate the performance of classifiers. In oncology, the ROC curve is widely used to evaluate the accuracy of tumor diagnosis and prediction models. Sensitivity represents the ratio of correctly identified samples that are actually positive, and specificity represents the ratio of correctly excluded samples that are actually negative. The ordinate of the ROC curve is the true positive rate (TPR, or sensitivity), and the abscissa is the false positive rate (FPR). TPR is the proportion of positive samples correctly classified, while FPR is the proportion of negative samples misclassified. Therefore, the ROC curve can show the trade-off between the sensitivity and specificity of the classifier under different thresholds. The area under the ROC curve (AUC) is commonly used in the evaluation of diagnostic tests. The AUC value range is generally between 0.5 and 1. The closer the AUC is to 1, the better the diagnostic effect of the variable in predicting the outcome. We used the XIANTAO tool to analyze the accuracy of ALKBH4 in the diagnosis and prediction model of gastric cancer.

### Gene expression analysis in GEPIA

We utilized the Gene Expression Profiling Interactive Analysis (GEPIA) database in our research to examine gene expression and correlations. GEPIA, accessible at http://gepia.cancer-pku.cn/index.html, incorporates data from 9736 tumors and 8587 normal samples retrieved from TCGA and GTEx cohorts. Specifically, we employed tumor and normal tissue datasets provided by GEPIA to establish ALKBH4 expression patterns. To assess correlations, we employed TCGA RNA-seq data and computed correlation coefficients using the Spearman method.

### TNM plot: differential gene expression analysis

TNM plot (https://tnmplot.com/analysis/) is a powerful online tool that enables comprehensive analysis of the TNM staging system in cancer research. This widely used system evaluates tumor severity based on parameters including tumor size, lymph node involvement, and distant metastasis. TNM plot offers several valuable features beyond TNM staging, such as survival analysis and identification of genes that influence patient prognosis. By analyzing the relationship between TNM staging and overall survival, researchers can better understand the molecular mechanisms underlying tumor progression. The TNM plot database contains a total of 56,938 samples, comprising 33,520 samples from 3180 gene chip studies (453 metastatic, 29,376 tumorigenic, and 3691 normal samples), 11,010 samples from TCGA (394 metastatic, 9886 tumorigenic, and 730 normal samples), 1193 samples from TARGET (1 metastatic, 1180 tumorigenic, and 12 normal samples), and 11,215 normal samples from GTEx. The Normal and Tumor analysis page utilizes gene chip data to perform in-depth analysis of ALKBH4 across all tissue types included in the database. TNM plot offers a convenient and user-friendly platform for conducting TNM staging analyses in cancer research. Its comprehensive features and extensive database provide valuable insights into tumor progression and patient prognosis.

### Survival analysis using Kaplan–Meier plotter

The Kaplan–Meier plotter (http://kmplot.com/analysis/) is a web-based tool for analyzing the impact of various genes on the survival of cancer patients. In this study, Kaplan–Meier plotter was used to analyze the effect of ALKBH4 expression on the prognosis and survival of patients with gastric cancer.

### Linkedomics database

The Linkedomics database (http://www.linkedomics.org/login.php) is an open multi-omics resource that contains data from 10 cancer cohorts from the Clinical Proteomics Tumor Analysis Consortium (CPTAC), as well as 32 cancer types from The Cancer Genome Atlas (TCGA). Linked Omics provides valuable gene set enrichment analysis (GSEA) and GO analysis (biological process) of ALKBH4, which can help shed light on its role in cancer development and progression.

The sample size calculation is based on efficacy analysis, including the expected effect size, expected significance level, and statistical efficacy (usually set at 80% or higher) from the pre-experiment. The G*Power software is employed to ensure that the sample size can provide sufficient power to detect pre-specified effect sizes with a certain degree of confidence.

### Statistical analysis

We used GraphPad Prism 8.2.1 (GraphPad, San Diego, California, USA) for data analysis and plotting. The data used were the mean ± standard deviation of at least three independent experiments. For comparison between the two sample groups, we performed analysis using the student *t*-test. The values presented were the mean ± standard deviation acquired from at least three independent experiments. In cases where there were multiple sample groups that required comparison, we utilized one-way ANOVA or two-way ANOVA, the latter being employed when there were independent variables between the groups. *P*-value < 0.05 was considered significant.

## Results

### ALKBH4 expression is upregulated and correlated with poor prognosis in GC

**Fig. 1:**
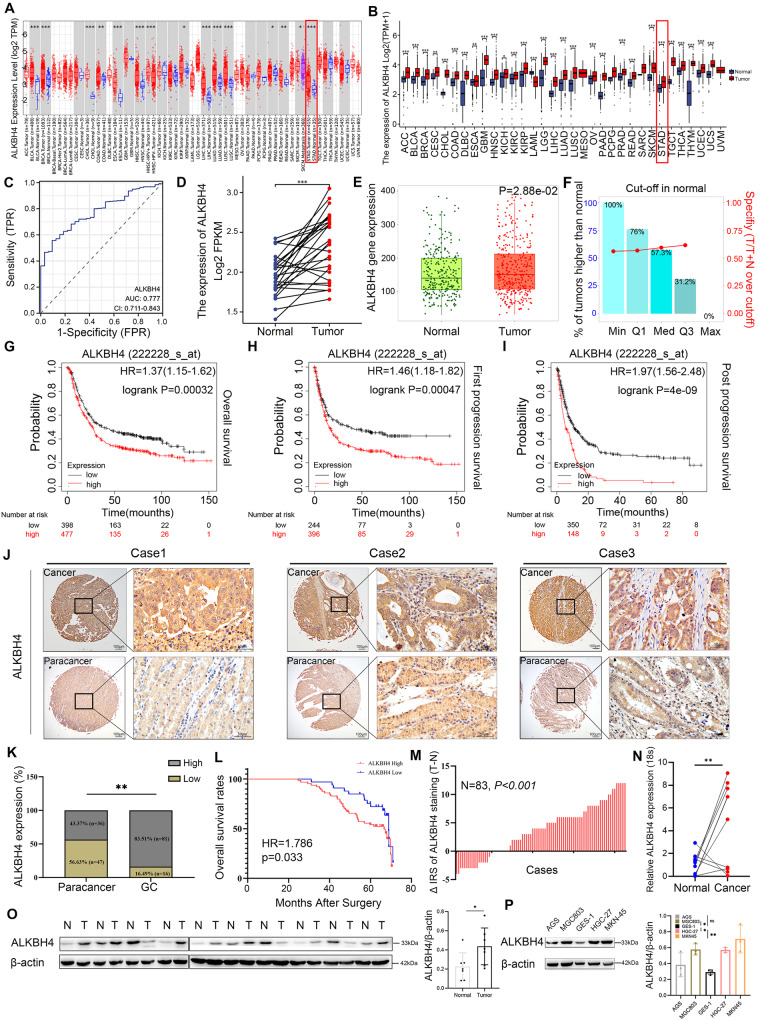
ALKBH4 expression is upregulated and correlated with poor prognosis in GC. **A** The expression of ALKBH4 mRNA in different types of tumor tissues and normal tissues in TIMER database. *p < 0.05, **p < 0.001, ***p < 0.0001. **B** Pan-cancer analysis of ALKBH4 mRNA expression was performed using TCGA and GTEx data. **C** ROC curve was used to evaluate the accuracy of ALKBH4 in the diagnosis of gastric cancer. **D** The differential expression of ALKBH4 in cancer and adjacent tissues was analyzed based on TCGA-STAD data. The data were analyzed by paired *t*-test. **E** Based on RNA-Seq data, ALKBH4 in gastric adenocarcinoma was analyzed in detail in Normal and Tumor. **F** The bar chart represents the proportion of tumor samples showing higher expression of ALKBH4 compared with normal samples at each quantile cut-off value (minimum, 1st quartile, median, 3rd quartile, maximum). The specificity is calculated by dividing the number of tumor samples by the sum of tumor and normal samples below each given cut-off value. When the fold change exceeds 1, use “more than” instead of “less than”. **G**–**I** Kaplan–Meier plotter was used to obtain OS, PF, and PPS curves of GC patients with low and high expression of ALKBH4. **J** Representative immunohistochemical staining of ALKBH4 in GC and normal tissues. **K** IHC statistical analysis of TMA showed the proportion of ALKBH4 expression in GC tissues and normal tissues. **L** OS Kaplan–Meier survival curve based on ALKBH4 expression. **M** ΔIRS of ALKBH4 based on TMA. **N** Quantitative analysis of ALKBH4 levels in GC tissues and normal tissues. The data were analyzed by paired *t*-test. **O** The expression of ALKBH4 protein in the lysate of fresh gastric cancer and adjacent normal tissues. β-actin was used as an internal control. T tumor tissue, N normal tissue. **P** To compare the differential expression of ALKBH4 protein in gastric cancer cells and normal gastric mucosal cells. β-actin was used as an internal control. Data are expressed as means ± standard errors of the means of three independent experiments, *P < 0.05, **P < 0.01.

We first detected the differential expression of nine ALKBH family proteins in gastric cancer tissues and normal tissues based on TCGA, TARGET, and GTEx databases, and found that in gastric cancer tissues, only ALKBH1, ALKBH4, ALKBH8, and FTO were highly expressed in gastric cancer tissues, among which FTO and ALKBH4 were differentially expressed in gastric cancer tissues and normal tissues, FTO and ALKBH4 were most significantly expressed in gastric cancer tissues and normal tissues (Fig. S1A). We also examined the relationship between ALKBH1, ALKBH4, ALKBH8, FTO, and survival prognosis of gastric cancer patients by the Kaplan–Meier method, and found that there was no statistically significant relationship between ALKBH1 and survival prognosis of gastric cancer patients, and both ALKBH4 and FTO were negatively correlated with the survival prognosis of gastric cancer patients, ALKBH8 has two transcripts, ALKBH8 (235610-at) was positively associated with the survival prognosis of gastric cancer patients, and ALKBH8 (235713-at) was negatively associated with the survival prognosis of gastric cancer patients (Fig. S1B). To further explore the relationship between the ALKBH family and gastric cancer, we examined the expression of nine ALKBH family proteins in cancerous and normal tissues using Western blot assays. We found that in 10 pairs of human gastric cancer and normal tissues, ALKBH1, ALKBH4, ALKBH8, and FTO were highly expressed in the gastric cancer tissues. Conversely, ALKBH2, ALKBH3, ALKBH5, ALKBH6, and ALKBH7 showed low expression in gastric cancer tissues. These findings are consistent with the results predicted by our bioinformatics analyses (Figs. 1O and S1C). However, ALKBH8 was highly expressed in only six pairs of gastric cancer tissues, while ALKBH4 was highly expressed in seven pairs, and FTO was highly expressed in all 10 pairs. The role of FTO in the progression of gastric cancer has been widely investigated; however, the role of ALKBH4 remains unclear. In summary, we selected ALKBH4 as the subject of our study. Through the TIMER online website and the XIANTAO tool, we analyzed the mRNA expression levels of ALKBH4 in various tumor types in TCGA and GTEx databases. The box plot clearly demonstrated a significant increase in ALKBH4 expression in most tumors compared to normal tissues (Fig. 1A, B). The ROC curve analysis of ALKBH4 in gastric adenocarcinoma demonstrated an AUC of 0.777 (95% CI: 0.711–0.843), indicating a moderate predictive performance. The AUC was calculated to evaluate the discrimination ability of ALKBH4 in distinguishing between gastric adenocarcinoma patients and healthy individuals (Fig. 1C). Transcriptome data in the TCGA-STAD project were downloaded and paired samples of cancer and adjacent normal tissues (*n* = 27) were analyzed. It was found that the expression of ALKBH4 in gastric cancer tissues was significantly higher than that in adjacent normal tissues (Fig. 1D). Furthermore, we verified that ALKBH4 was significantly overexpressed in gastric cancer tissues by TNM plot online database (Fig. 1E, F). Kaplan–Meier survival analysis showed that high expression of ALKBH4 may lead to shorter overall survival, first prognostic survival, and post-progression survival in patients with gastric cancer (Fig. 1G–I). IHC staining to examine the expression of ALKBH4 in a tissue microarray consisting of 97 GC samples and 83 normal samples. The IHC staining results clearly demonstrated strong ALKBH4 staining in GC tissues, whereas adjacent normal tissues exhibited weak staining (Fig. 1J). The staining results revealed a statistically significant difference in ALKBH4 expression levels between GC tissues and normal tissues (Fig. 1K). Moreover, gastric cancer patients with high ALKBH4 expression exhibited a poor overall survival rate (Fig. 1L). ΔIRS (Immunohistochemical reaction score) of ALKBH4 staining also confirmed that ALKBH4 was highly expressed in gastric cancer tissues (Fig. 1M). A total of 10 paired fresh tumor tissues and adjacent normal tissues were collected from patients with gastric cancer at the Affiliated Hospital of Xuzhou Medical University. Through qPCR experiments, we observed significant upregulation of ALKBH4 at mRNA levels in gastric cancer tumors (Fig. 1N). The Western blot analysis demonstrated that ALKBH4 protein expression was notably higher in gastric cancer cell lines (AGS, MGC803, HGC-27, and MKN45) compared to normal mucosal epithelial cells (GES-1). Densitometry analysis confirmed a statistically significant upregulation of ALKBH4 in tumor cells when compared to normal cells (*p* < 0.05) (Fig. 1P). Furthermore, ALKBH4 expression was higher in gastric cancer cell lines MKN45, HGC-27, and AGS compared to fibroblasts (Fig. S1D). Thus, our findings suggest that ALKBH4 is overexpressed in GC, indicating its potential as a diagnostic and prognostic biomarker in this malignancy.

### ALKBH4 promotes gastric cancer cell proliferation and inhibits gastric cancer cell pyroptosis in vitro

**Fig. 2:**
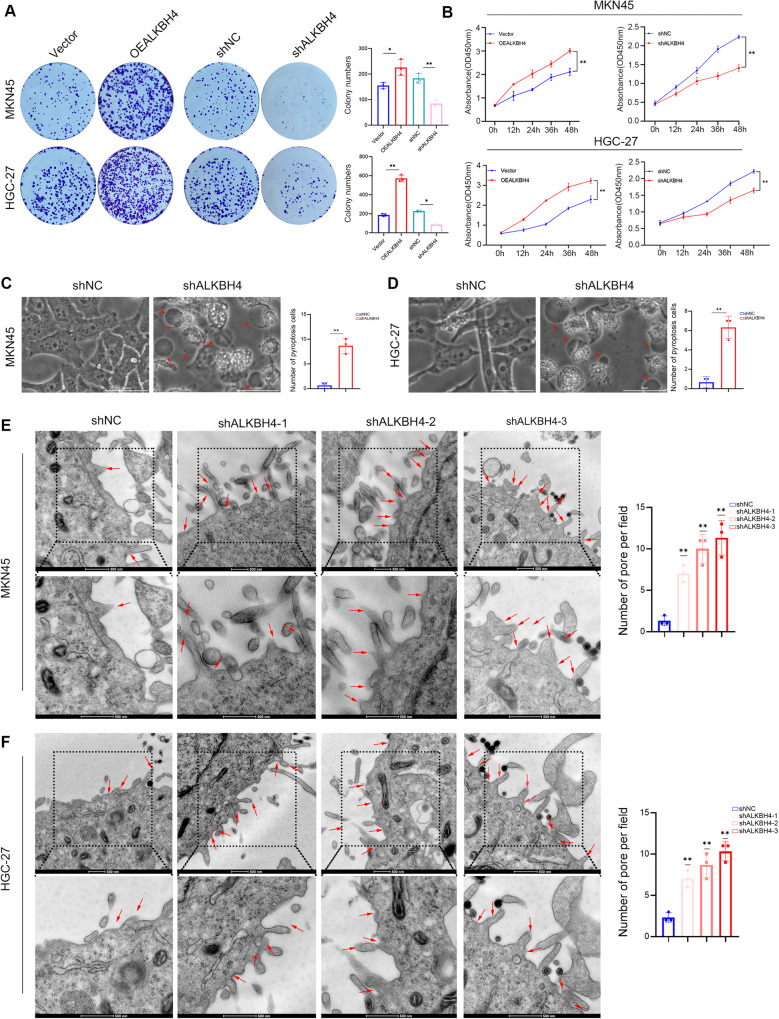
ALKBH4 promotes gastric cancer cell proliferation and inhibits gastric cancer cell pyroptosis in vitro. **A** Colony formation assay of gastric cancer cells treated with ALKBH4 overexpression or shALKBH4. **B** The proliferation curve of gastric cancer cells treated with ALKBH4 overexpression or shALKBH4. **C**, **D** Pyroptosis morphology of cells was observed under electron microscope (Red arrows indicate bubble-like structures). Scale bar is 50 μm. **E**, **F** Representative images of MKN45 and HGC-27 cells transfected with ALKBH4 shRNAs or control shRNAs were analyzed by transmission electron microscopy. The red arrow indicates the pores on the plasma membrane. The scale is 500 nm. Data are expressed as means ± standard errors of the means of three independent experiments, *P < 0.05, **P < 0.01.

In order to determine whether the expression level of ALKBH4 regulates the proliferation of gastric cancer cells, we demonstrated that ALKBH4 overexpression led to a significantly higher number of proliferating cells compared to the vector control group in both MKN45 and HGC-27 cell lines by plate cloning experiments. Conversely, knockdown of ALKBH4 expression can significantly inhibit the proliferation of gastric cancer cells. The results of the CCK8 assay further substantiated these findings. The overexpression of ALKBH4 was found to enhance the proliferation activity of gastric cancer cells, while the knockdown of ALKBH4 was observed to inhibit the proliferation activity of gastric cancer cells (Fig. 2A, B). Further observation of gastric cancer cells after ALKBH4 knockdown under an electron microscope revealed signs of pyroptosis. Morphologically, ALKBH4 knockdown cells showed large bubbles and cell swelling from the plasma membrane. Quantitative analysis revealed a higher incidence of pyroptosis markers in gastric cancer cells following ALKBH4 knockdown compared to the control group (Fig. 2C, D). TEM images revealed an increase in pore formation on the plasma membrane of cells subjected to ALKBH4 knockdown when compared to control cells. The pores on the plasma membrane are formed by the generation of GSDME-N, indicating that the number of pyroptosis cells increased after ALKBH4 knockdown (Fig. 2E, F). The above data suggest that ALKBH4 may affected cell proliferation by reducing pyroptosis.

### ALKBH4 negatively regulates the expression of GSDME in GC

**Fig. 3:**
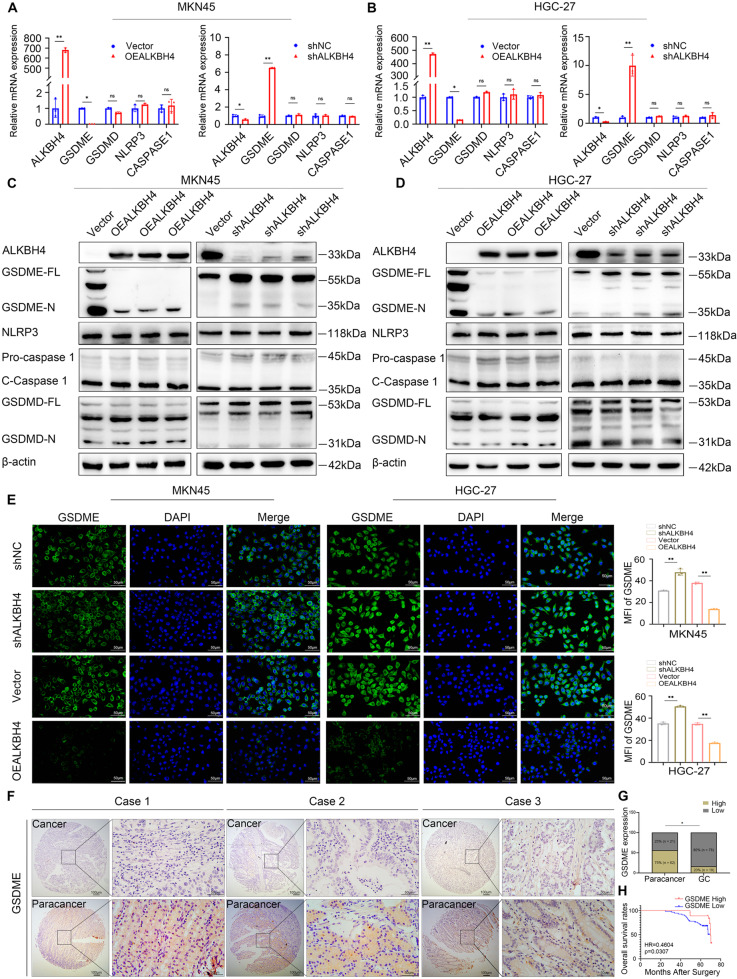
ALKBH4 negatively regulates the expression of GSDME in GC. **A**, **B** The mRNA levels of GSDME, NLRP3, GSDMD, Caspase 1, and GSDMD were analyzed by qPCR after overexpression and knockdown of ALKBH4 in MKN45 and HGC-27 cells. **C**, **D** Western blot was used to detect the protein expression levels of GSDME, NLRP3, GSDMD, Caspase 1, and GSDMD after overexpression and knockdown of ALKBH4 in MKN45 and HGC-27 cells. **E** Immunofluorescence assay was employed to analyze the impact of ALKBH4 overexpression or ALKBH4 knockdown on the expression level of GSDME in gastric cancer cells MKN45 and HGC-27. Quantitative analysis of the fluorescence intensity showed on the right side. **F** Representative immunohistochemical staining of GSDME in GC and normal tissues. **G** Quantitative statistics on the expression of GSDME in 97 cases of gastric cancer tissues and 83 cases of normal gastric tissues. **H** OS Kaplan–Meier survival curve based on ALKBH4 expression. Data are expressed as means ± standard errors of the means of three independent experiments, *P < 0.05, **P < 0.01.

To elucidate the underlying mechanism of how ALKBH4 inhibits pyroptosis in gastric cancer cells, we evaluated the impact of ALKBH4 expression on key targets (GSDME, GSDMD, NLRP3, Caspase 1) of the pyroptosis pathway using qPCR and western blot experiments. Notably, overexpression of ALKBH4 resulted in a significant decrease in mRNA and protein levels of GSDME. Conversely, inhibition of ALKBH4 resulted in a notable increase in the mRNA and protein expression of GSDME. Nevertheless, the overexpression and knockdown of ALKBH4 demonstrated no significant impact on the mRNA and protein expression levels of GSDMD, NLRP3, and Caspase 1 (Fig. 3A–D). These findings indicated that ALKBH4 has the potential to enhance pyroptosis by suppressing GSDME expression, while not affecting crucial molecules within alternative pyroptosis pathways. Furthermore, ALKBH4 overexpression led to the inhibition of GSDME expression, whereas ALKBH4 knockdown resulted in an increase in GSDME expression levels. The quantitative results of mean fluorescence intensity (MFI) were shown on the right side (Fig. 3E). Subsequently, to validate the clinical relevance of GSDME, we performed immunohistochemical staining on gastric cancer tissues. Our results revealed that GSDME expression was significantly lower in tumor tissues compared to adjacent normal tissues (Fig. 3F). The results of quantitative analysis on GSDME expression in 97 cases of gastric cancer and 83 cases of normal gastric tissues indicated that GSDME exhibited high expression levels in gastric cancer tissues while being expressed at low levels in adjacent normal tissues (Fig. 3G). Moreover, survival analysis demonstrated that patients with low GSDME expression exhibited a poorer prognosis (Fig. 3H). To summarize, our findings emphasize the role of ALKBH4 as a negative regulator of GSDME expression, resulting in the suppression of pyroptosis.

### ALKBH4 mediates the transcriptional activation of GSDME by reducing the modification level of H3K4me3 in the promoter region of GSDME

**Fig. 4:**
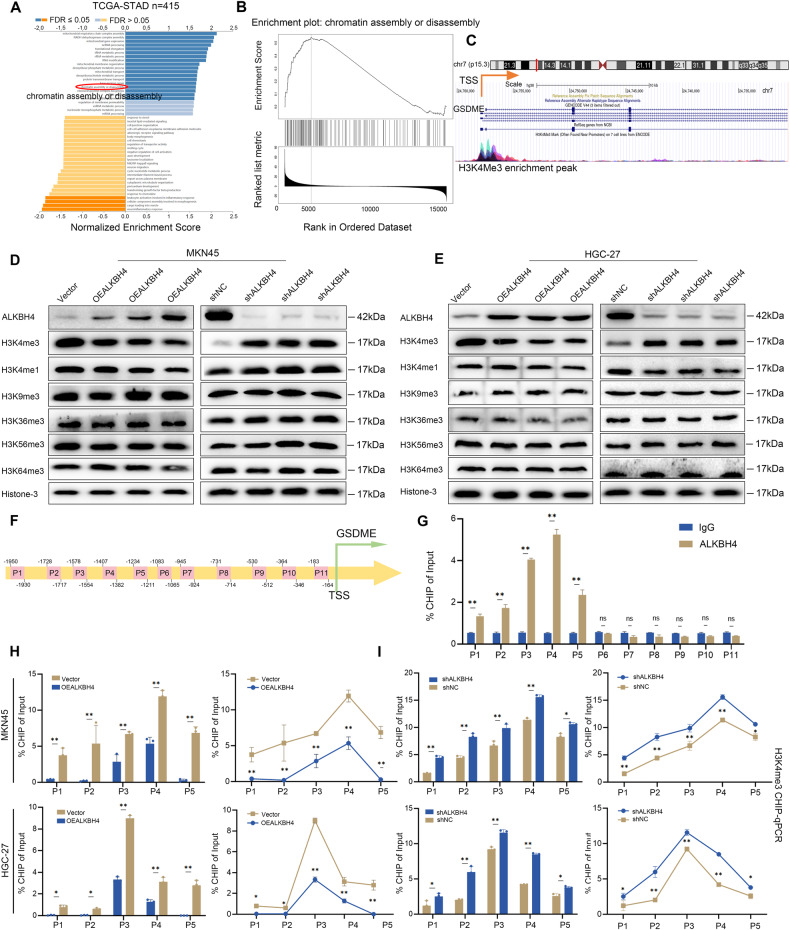
ALKBH4 mediates the transcriptional activation of GSDME by reducing the modification level of H3K4me3 in the promoter region of GSDME. **A**, **B** GO biological process analysis and GSEA of differential genes involved in ALKBH4. **C** Enrichment of H3K4me3 binding peak in GSDME promoter region. **D**, **E** Protein expression levels of H3K4me3, H3K4me1, H3K9me3, H3K36me3, H3K56me3, and H3K64me3 were assessed using Western blot analysis following ALKBH4 overexpression and knockdown in GC cells. **F** The spatial distribution of 11 primer sets targeting the truncated fragments of the GSDME promoter was determined. **G** ChIP results of anti-IgG and anti-ALKBH4 antibodies in MKN45 cells. **H**, **I** To assess the binding efficiency of H3K4me3 to the GSDME promoter region, CHIP assay was performed following ALKBH4 overexpression and knockdown in MKN45 and HGC-27 cells. Data are expressed as means ± standard errors of the means of three independent experiments, *P < 0.05, **P < 0.01.

GO analysis results revealed that ALKBH4 was positively involved in chromatin assembly and disassembly (Fig. 4A). Furthermore, we performed Gene Set Enrichment Analysis (GSEA) and confirmed that ALKBH4 amplification was indeed positively correlated with chromatin assembly and depolymerization in gastric cancer (Fig. 4B). These results suggest that ALKBH4 may be involved in histone modification. Our analysis of the UCSC online database (https://genome.ucsc.edu/index.html) revealed a significant enrichment peak of H3K4me3 in the promoter region of GSDME (Fig. 4C). Our results showed that overexpression of ALKBH4 specifically inhibited the modification of H3K4me3, while other histone markers such as histone H3K4me1, H3K9me3, H3K36me3, H3K56me3 and H3K64me3 were not affected. Conversely, the knockdown of ALKBH4 resulted in the promotion of H3K4me3 modification (Fig. 4D, E). To further elucidate the relationship between ALKBH4 and GSDME, a set of 11 primers was meticulously designed to specifically target the truncated fragment of the GSDME promoter (Fig. 4F). Through ChIP assays, it was observed that ALKBH4 exhibits binding affinity to the GSDME promoter region spanning from P1 to P5 (Fig. 4G). Subsequently, we conducted ChIP assays following ALKBH4 overexpression. Our results revealed a significant decrease in the binding efficiency of H3K4me3 to the GSDME promoter region, suggesting that ALKBH4 negatively regulated the transcriptional activation of GSDME by demethylating histone modifications in the GSDME promoter region (Fig. 4H). Conversely, the knockdown of ALKBH4 expression increased the binding efficiency of H3K4me3 to the GSDME promoter region (Fig. 4I). Taken together, these findings confirmed that ALKBH4 inhibits GSDME transcriptional activation by inhibiting the H3K4me3 modification level in the GSDME promoter region.

### ALKBH4 affects the sensitivity of GC cells to 5-FU in vitro

**Fig. 5:**
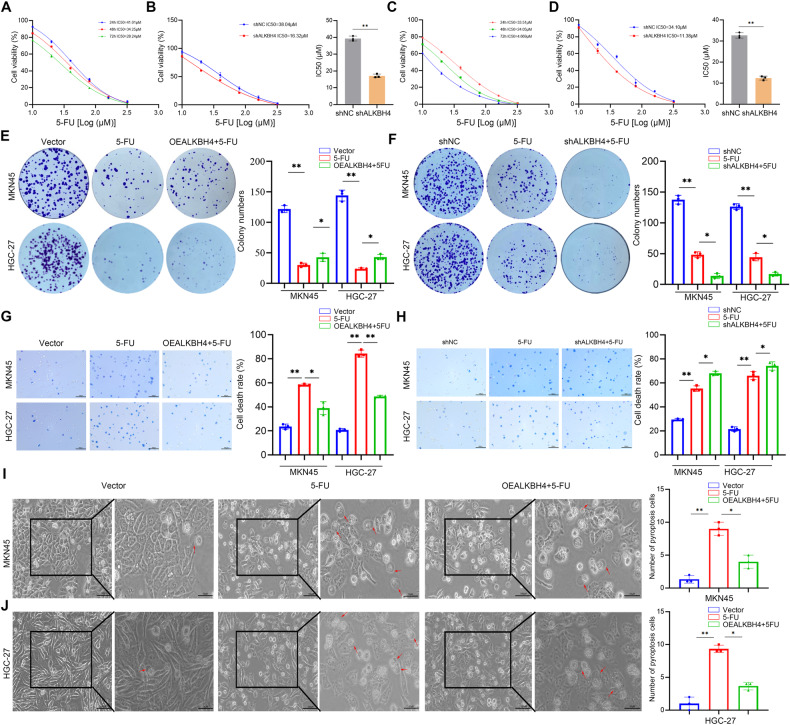
ALKBH4 affects the sensitivity of GC cells to 5-FU in vitro. **A**, **C** The IC50 (24 h, 48 h, 72 h) of gastric cancer cells MKN45 and HGC-27 was detected by CCK8 assay. **B**, **D** CCK8 experiments showed that ALKBH4 knockdown reduced the IC50 value of 5-FU in gastric cancer cells. **E** The effect of overexpression of ALKBH4 and 5-FU on the proliferation of gastric cancer cells was verified by colony formation assay. **F** The effect of knockdown of ALKBH4 and 5-FU treatment on the proliferation of gastric cancer cells was verified by colony formation assay. **G** Trypan blue staining was used to detect the mortality of gastric cancer cells after overexpression of ALKBH4 and 5-FU treatment. **H** Trypan blue staining was used to detect the mortality of gastric cancer cells after knockdown of ALKBH4 and 5-FU treatment. **I** The morphological changes of pyroptosis after overexpression of ALKBH4 and 5-FU treatment were observed under a microscope. **J** The morphological changes of pyroptosis after knockdown of ALKBH4 and 5-FU treatment were observed under a microscope. Data are expressed as means ± standard errors of the means of three independent experiments, *P < 0.05, **P < 0.01.

Pyroptosis is a regulated form of cell death and has been shown to be related to the response of tumor cells to chemotherapeutic drugs [12, 44–46]. Previous studies have demonstrated that treatment with 5-FU induces pyroptosis in gastric cancer cells, characterized by swelling and rupture of the cell membrane, and identified GSDME as a mediator of Caspase 3-dependent apoptosis to pyroptosis conversion in gastric cancer cells [47]. We detected the IC50 values of gastric cancer cell lines MKN45 and HGC-27 to 5-FU by CCK8 assay (Fig. 5A, C). After knocking down ALKBH4, we found that the IC50 values of both cells were decreased, suggesting that the decreased expression of ALKBH4 may promote the sensitivity of gastric cancer cells to 5-FU (Fig. 5B, D). Subsequent plate cloning experiments showed that the cell proliferation ability of MKN45 and HGC-27 GC cells was significantly inhibited after 5-FU treatment, and the overexpression of ALKBH4 rescued the inhibitory effect of 5-FU on the proliferation of gastric cancer cells (Fig. 5E). In addition, knockdown of ALKBH4 aggravated the inhibitory effect of 5-FU on the proliferation of gastric cancer cells (Fig. 5F). The consistent results were also obtained by the trypan blue experiment. After GC cells were treated with 5-FU, the cell death rate was significantly increased, and overexpression of ALKBH4 led to a decrease in the 5-FU-induced cell death rate (Fig. 5G). Knockdown of ALKBH4 promoted 5-FU-induced cell death (Fig. 5H). Furthermore, we observed an elevation in the occurrence of cell pyroptosis (microscopically characterized by cell membrane swelling) following the administration of 5-FU, and the overexpression of ALKBH4 mitigated 5-FU-induced cell pyroptosis (Fig. 5I, J). These results suggest that the knockdown of ALKBH4 increases the chemosensitivity of gastric cancer cells by promoting 5-FU-induced pyroptosis.

### ALKBH4 reduced the chemosensitivity of gastric cancer cells to 5-FU through GSDME

**Fig. 6:**
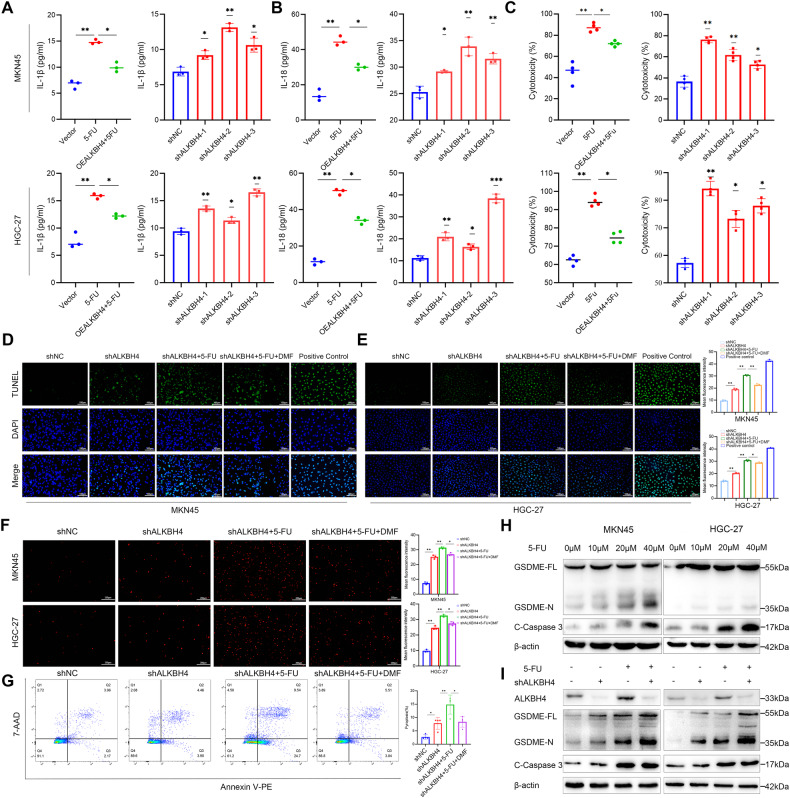
ALKBH4 reduced the chemosensitivity of gastric cancer cells to 5-FU through GSDME. **A** The expression of IL-1β and IL-18 in GC cells overexpressing ALKBH4 after 5-FU treatment was analyzed by ELISA. **B** ELISA was used to analyze the expression of IL-1β and IL-18 in the supernatant of GC cells that underwent ALKBH4 knockdown. **C** We examined the effect of ALKBH4 overexpression on 5-FU-induced cytotoxicity and evaluated the effect of knockdown of ALKBH4 on the cytotoxicity of MKN45 and HGC-27 cells. **D**, **E** Chromatin integrity was detected by TUNEL. Quantitative analysis of the fluorescence intensity showed on the right side. **F** PI staining of MKN45 and HGC-27 cells was examined by fluorescence microscope. Quantitative analysis of the fluorescence intensity showed on the right side. **G** Flow cytometry was used to detect cell pyroptosis. **H** Western blot was used to detect the protein expression of GSDME and Cleaved-Caspase 3 after gastric cancer cells were treated with different concentrations of 5-FU. C-Caspase 3: Cleaved-Caspase 3. **I** The protein expression of GSDME and Cleaved-Caspase 3 was detected by western blot after 5-FU treatment of ALKBH4 knockdown gastric cancer cells. Data are expressed as means ± standard errors of the means of three independent experiments, *P < 0.05, **P < 0.01.

Pyroptosis induces the cleavage of GSDME, leading to the generation of GSMDE-N fragments. The oligomerization of the N-terminal domain subsequently facilitates the formation of cell membrane pores, thereby facilitating the release of cellular contents such as LDH, IL-1β, and IL-18 [48]. The ELISA results demonstrated a significant increase in the levels of IL-1β and IL-18 in the cell supernatant following treatment with 5-FU, as compared to the untreated group. Notably, the overexpression of ALKBH4 in conjunction with 5-FU treatment for 24 h exhibited a reversal in the heightened levels of IL-1β and IL-18 in the cell supernatant induced by 5-FU. Conversely, the knockdown of ALKBH4 led to an elevation in the levels of IL-1β and IL-18 in the supernatant (Fig. 6A, B). Cytotoxicity was detected by LDH release kit. Compared with the untreated group, the cytotoxicity level increased after 5-FU treatment. However, overexpression of ALKBH4 effectively inhibited 5-FU-induced cytotoxicity. Knockdown of ALKBH4 significantly increased cytotoxicity (Fig. 6C). To investigate the specific mechanism of ALKBH4’s effect on 5-FU chemosensitivity in gastric cancer cells, we conducted TUNEL and PI immunofluorescence experiments and found that knockdown of ALKBH4 promoted 5-FU-induced cell death. Moreover, we administered dimethyl fumarate (DMF), a pyroptosis inhibitor for GSDME/GSDMD, to the cells and noted a reduction in cell mortality, indicating that ALKBH4 mediates the sensitivity of gastric cancer cells to 5-FU through GSDME (Fig. 6D–F). Subsequently, we used flow cytometry to detect the staining of two dyes, 7-AAD, and Annexin V-PE, and found that the downregulation of ALKBH4 increased the double staining proportion of both dyes in MKN45 cells. Furthermore, compared to the group where only ALKBH4 was downregulated, the double staining proportion of 7-AAD and Annexin V-PE was higher in the group treated with a combination of downregulating ALKBH4 and 5-FU. However, DMF reversed the decrease of 7-AAD and Annexin V-PE double staining ratio induced by ALKBH4 and 5-FU combined treatment (Fig. 6G). Subsequently, we conducted Western blot analysis to confirm that an escalation in the dosage of 5-FU leads to the activation of Caspase 3, resulting in the cleavage of GSDME and subsequently generating a higher quantity of GSDME-N fragments (Fig. 6H). Significantly, our findings indicate that the suppression of ALKBH4 resulted in an upregulation of GSDME expression and an increase in GSDME-N levels even without the administration of 5-FU. However, this knockdown did not impact the activation of Caspase 3. These results suggest that ALKBH4 promotes the expression of GSDME in a manner independent of Caspase 3. Furthermore, when ALKBH4 was knocked down in combination with 5-FU treatment, it induced enhanced GSDME cleavage and elevated levels of GSDME-N (Fig. 6I). Importantly, we demonstrate that ALKBH4 knockdown amplifies GSDME transcriptional activation, thereby facilitating chemotherapeutic drug-induced pyroptosis and ultimately enhancing the chemosensitivity of gastric cancer cells.

### ALKBH4 knockdown promotes 5-FU-mediated inhibition of gastric cancer proliferation in vivo

**Fig. 7:**
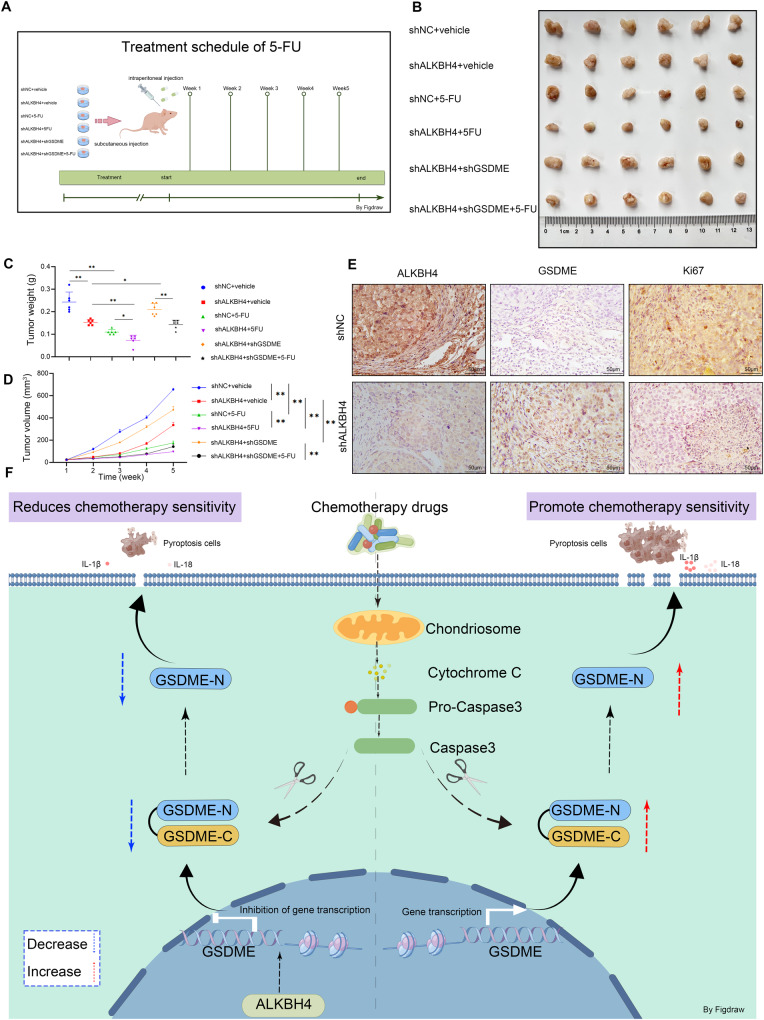
Knockdown of ALKBH4 promotes 5-FU-mediated inhibition of gastric cancer proliferation in vivo. **A** We inoculated immunodeficient mice with MKN45 cells stably expressing the target gene. Chemotherapeutic drugs were administered via intraperitoneal injection every three days, and tumor volume was measured once a week. **B** Subcutaneous xenografts in nude mice constructed with MKN45 cells. (*n* = 6 mice per group). **C**, **D** Tumor growth curves and weight of the tumors for indicated groups (*n* = 6 mice per group). **E** Immunohistochemical analysis of mouse tumor tissues were performed with anti-ALKBH4, anti-GSDME, and anti-Ki67 antibodies. **F** The mechanism of ALKBH4 mediates the transcriptional activation of GSDME affects the pyroptosis of gastric cancer cells and thus reduces the chemosensitivity of gastric cancer cells. Data are expressed as means ± standard errors of the means of three independent experiments, *P < 0.05, **P < 0.01.

We injected immunodeficient mice with MKN45 cells that had stable expression of the target gene. Chemotherapeutic drugs were administered through intraperitoneal injection every three days, and tumor volume was measured once a week (Fig. 7A). The knockdown of ALKBH4 inhibited the proliferation and retarded the growth of gastric cancer. When 5-FU alone was used to treat nude mice, it also inhibited the progression of gastric cancer and retarded the growth rate of gastric cancer. The inhibitory effect of 5-FU treatment alone on gastric cancer was higher than the inhibitory effect of gastric cancer by knocking down ALKBH4. The combination of 5-FU and ALKBH4 knockdown demonstrated the most efficacious therapeutic outcome in this experiment, with a notable reduction in gastric cancer proliferation and progression. Conversely, GSDME knockdown negated the gastric cancer growth inhibitory effect induced by ALKBH4 downregulation and 5-FU treatment (Fig. 7B–D). Furthermore, ALKBH4, GSDME, and Ki67-specific antibodies were employed for immunohistochemical staining of transplanted tumors. The results of immunohistochemistry experiments demonstrated that the proportion of GSDME staining increased and Ki67 staining decreased in xenograft tumors following ALKBH4 knockdown (Fig. 7E), thereby providing further evidence that ALKBH4 knockdown inhibits gastric cancer proliferation. The mechanism of action of ALKBH4 in gastric cancer involves reducing the level of H3K4me3 modification in the promoter region of GSDME by acting as a demethylase, leading to the inhibition of GSDME transcriptional activation. This ultimately suppresses 5-FU-induced cellular pyroptosis and decreases the sensitivity of gastric cancer to 5-FU chemotherapy (Fig. 7F).

## Discussion

GC, a solid tumor known for its complexity and peril, poses significant challenges to clinical treatment [1, 49]. Over the years, 5-FU has been extensively employed as the initial medication for advanced GC [49]. Nonetheless, its effectiveness in conjunction with other drugs to enhance the prognosis of GC patients remains limited [50]. The primary cause for the failure of 5-FU-based chemotherapy regimens in gastric cancer treatment can be attributed to drug resistance, leading to subsequent recurrence or metastasis [2]. Thus, there is an urgent need to comprehensively elucidate the molecular and cellular mechanisms underlying the occurrence and progression of gastric cancer, in order to establish more efficacious interventions for its treatment.

The ALKBH family of proteins has been the subject of extensive research, with ALKBH4 emerging as a particularly intriguing member. This protein has been shown to possess a range of biological functions, including the ability to remove the methyl group from monomethylated lysine or N6-methyl-2′-deoxyadenosine (6-mdA) [51]. ALKBH4 plays a pivotal role in the early development of mice. Experiments involving the targeted deletion of the ALKBH4 gene in young mice have demonstrated that this disruption can lead to abnormalities in spermatogenesis [52]. A recent study also demonstrated that ALKBH4 mutant mice exhibited normal behavior, with the exception of higher levels of germ cell apoptosis [28]. These findings indicate a potential role for ALKBH4 in both embryogenesis and spermatogenesis. Furthermore, ALKBH4 is located within the nucleus of germ cells, suggesting a significant involvement of this protein in the processes of mammalian embryogenesis and gene transcription [19, 28]. Our animal experiments results have been consistent with the results of in vitro cellular experiments, suggesting that ALKBH4 acts as a tumor-promoting factor in mice, and knockdown of ALKBH4 significantly inhibited the proliferative capacity of gastric cancer cells. However, we did not detect the apoptosis level of germ cells in experimental mice, and this part of the study needs to be carried out at a deeper level. In addition, the interaction of ALKBH4 with monomethylated lysine (K84me1) in actin in the cytoplasm of human cells has been observed. ALKBH4 was found to be elevated at contractile loops and midbody structures, suggesting that ALKBH4 may be a novel factor in cell division [19]. Furthermore, ALKBH4 is a demethylase of K84me actin that regulates actin-myosin II interaction [40]. It has been reported that ALKBH4 is overexpressed in head and neck squamous cell carcinoma (HNSCC) [27] and non-small-cell lung cancer (NSCLC). Depletion of ALKBH4 significantly impedes NSCLC cell proliferation by inducing G1 phase cell cycle arrest during tumor growth in vivo [53]. In contrast, in colorectal cancer (CRC), ALKBH4 acts as a tumor suppressor, inhibiting the invasive and metastatic ability of CRC cells by inhibiting epithelial-mesenchymal transition (EMT) [54]. In our study, we found high expression of ALKBH4 in gastric cancer and its positive correlation with poor prognosis of GC patients through the TCGA database. And our findings show that elevated ALKBH4 expression can effectively enhance the proliferation and advancement of gastric cancer both in vitro and in vivo. Furthermore, we have identified the pivotal role played by ALKBH4 in increasing the response of gastric cancer cells towards 5-FU chemotherapy. Downregulating the expression of ALKBH4 markedly enhanced the susceptibility of gastric cancer cells to 5-FU, leading to the suppression of gastric cancer cell proliferation.

Moreover, it has been demonstrated that chemotherapeutic agents can induce the activation of Caspase 3 by damaging DNA, with the activated Caspase 3 subsequently mediating the cleavage of GSDME. Western blot experiments were conducted to confirm that 5-FU was able to activate Caspase 3, which contributed to the cleavage of GSDME, leading to the production of more GSDME-N fragments. Moreover, the GSDME-N fragments produced by cleavage exhibited a gradual increase in concentration with the elevation of 5-FU concentration. In contrast to previous studies, the knockdown of ALKBH4 was found to promote GSDME expression without affecting caspase 3 activation, resulting in elevated levels of GSDME-N in the absence of 5-FU. The results indicated that ALKBH4 may promote GSDME expression in a caspase 3-independent manner. Furthermore, the production of GSDME-N terminals was found to be greater when ALKBH4 was knocked down and co-treated with 5-FU than when ALKBH4 was knocked down alone or treated with 5-FU alone. Consequently, our study offers a novel approach to the inhibition of tumor development by utilizing cellular pyroptosis. It is important to highlight that GSDME has emerged as a promising target for enhancing chemosensitivity, garnering significant attention as a key player in the pathways leading to chemotherapy-induced cell death [55]. Currently, it has been verified that chemotherapy-induced pyroptosis enhances the cytotoxicity against malignant tumor cells. Studies have observed a positive correlation between increasing concentrations of cisplatin and doxorubicin and the levels of NLRP3 inflammasome protein and Caspase 1 in human mesothelioma cell line Hmeso, which eventually leads to increased pyroptosis [56]. However, the initiation of pyroptosis requires a high level of GSDME expression, and the proportion of GSDME in tumor cells is low due to epigenetic gene silencing [57–59]. For instance, low expression of GSDME has been found in 52% of early-stage gastric cancer cases [59]. Nevertheless, this also provides an opportunity for chemo-sensitization by enhancing the expression of pyroptosis-associated proteins like GSDME during chemotherapy. In malignant tumors, the low expression of GSDME related to pyroptosis can make melanoma MeWo cells obtain resistance to etoposide. The increased expression of GSDME in tumor cells may make it more sensitive to chemotherapy [60]. The high expression of GSDME was also observed in breast cancer MCF-7 cells. It has been confirmed that the decrease of GSDME expression in MCF-7 cells may contribute to the acquired resistance to paclitaxel [12]. Therefore, increasing the expression of pyroptosis-related proteins and enhancing pyroptosis of tumor cells are conducive to improving chemotherapy sensitivity and reversing chemotherapy resistance. Our findings indicate that the inhibition of ALKBH4 expression may enhance the sensitivity of gastric cancer cells to 5-FU by increasing the expression level of GSDME in gastric cancer.

Furthermore, ALKBH4 has been demonstrated to interact with chromatin-associated proteins and/or proteins involved in transcription [61]. Studies have demonstrated that ablation of ALKBH4 led to alterations in the expression levels of a large number of proteins, including the pronounced attenuation of the expression of HSPB1 and GSTP1. Mechanistic studies demonstrated that the loss of ALKBH4 resulted in elevated expression of DNMT1 protein, which in turn led to augmented promoter methylation and the subsequent epigenetic silencing of HSPB1 and GSTP1 genes [62]. In addition, it has been reported that ALKBH4 inhibits H3K4me3 modification by binding to WDR5 in CRC [54]. However, ALKBH4 regulates gastric cancer progression through demethylation has not been reported. Interestingly, we observed that the H3K4me3 signal was enriched in the GSDME promoter, suggesting that chromatin remodeling is involved in the regulation of GSDME expression. Through ChIP analysis, we demonstrated that ALKBH4 inhibits the transcriptional activation of GSDME by reducing the level of H3K4me3 in its promoter region, thereby inhibiting pyroptosis.

Although studies have confirmed that ALKBH4 is involved in the negative regulation of GSDME expression in gastric cancer cells, there are still many unknowns in other related pathways that ALKBH4 regulates pyroptosis. Therefore, it is necessary to carry out more studies to explore the potential mechanism of ALKBH4. Taken together, our study demonstrates the potential value of ALKBH4 as a novel therapeutic target for improving chemosensitivity by regulating the pyroptosis process of gastric cancer cells.

## Conclusion

In summary, our results confirm that ALKBH4 attenuates the sensitivity of gastric cancer cells to 5-FU treatment by inhibiting GSDME transcription. These findings may provide new insights into the treatment of GC and improve the chemosensitivity of gastric cancer.

### Supplementary information

Supplementary material

original experimental data

## Data Availability

The original contributions presented in the study are included in the article, further inquiries can be directed to the corresponding authors.
