# Supplementary material for: ALKBH4 impedes 5-FU Sensitivity through suppressing GSDME induced pyroptosis in gastric cancer
Source: Cell Death Dis. 2024 Jun 20;15(6):435. doi: 10.1038/s41419-024-06832-1 (PMC11189908; doi:10.1038/s41419-024-06832-1)
Supplement: Supplementary file 1 — Supplementary material [file 41419_2024_6832_MOESM1_ESM.docx]

Supplementary Figure Legends:


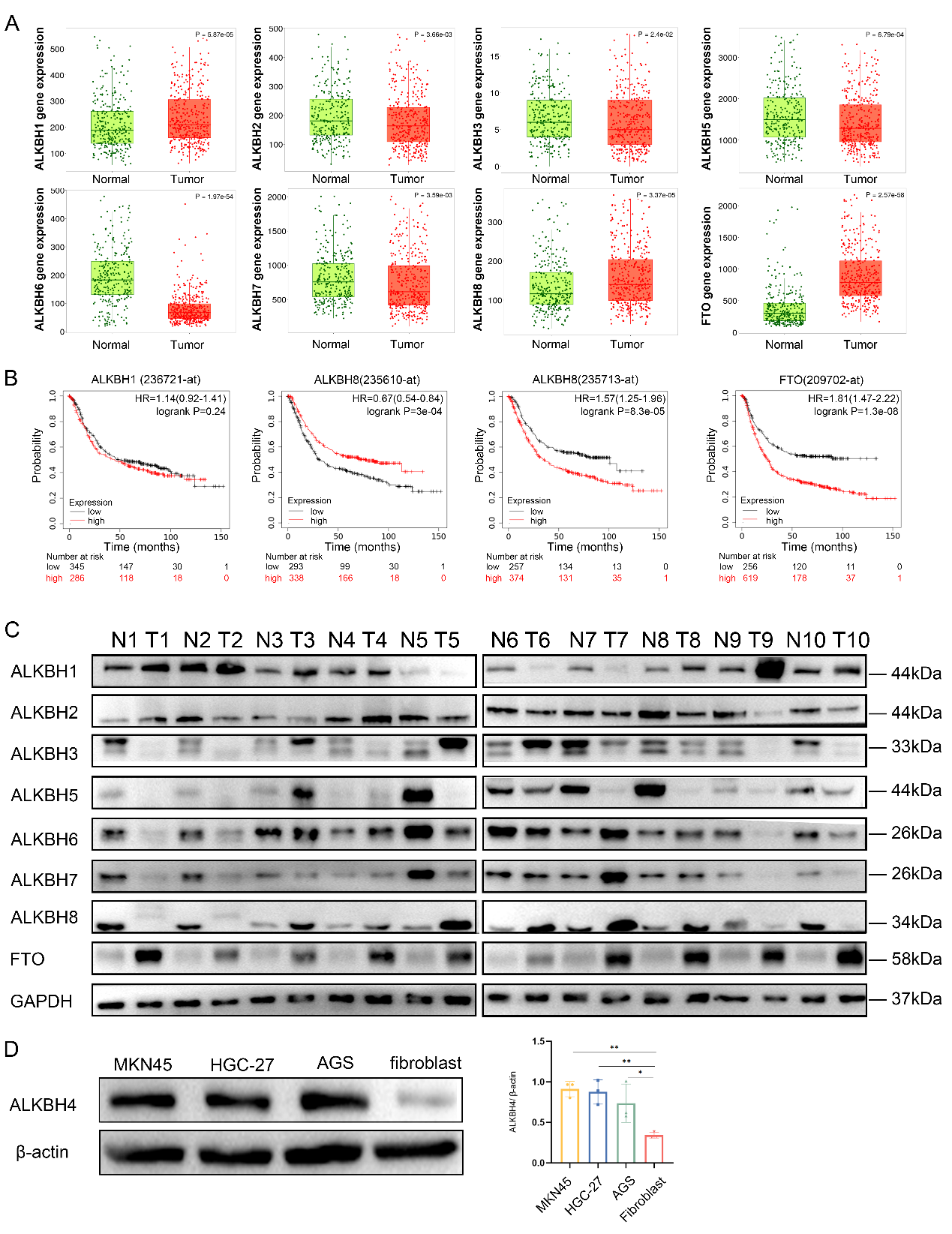


Figure S1: The relationship between the ALKBH family and gastric cancer. A: A comparative analysis of the differential expression of ALKBH1, ALKBH2, ALKBH3, ALKBH5, ALKBH6, ALKBH7, ALKBH8, and FTO in gastric cancer tissues and normal tissues. B: Kaplan-Miere analysis of ALKBH1, ALKBH8, FTO and survival prognosis of gastric cancer patients. C: Analysis of ALKBH family expression in gastric cancer tissues and normal tissues. D: To compare the differential expression of ALKBH4 protein in gastric cancer cells and fibroblast cells. *P < 0.05, **P < 0.01
