# Supplementary material for: ALKBH4 impedes 5-FU Sensitivity through suppressing GSDME induced pyroptosis in gastric cancer
Source: Cell Death Dis. 2024 Jun 20;15(6):435. doi: 10.1038/s41419-024-06832-1 (PMC11189908; doi:10.1038/s41419-024-06832-1)

Figure 1O

ALKBH4

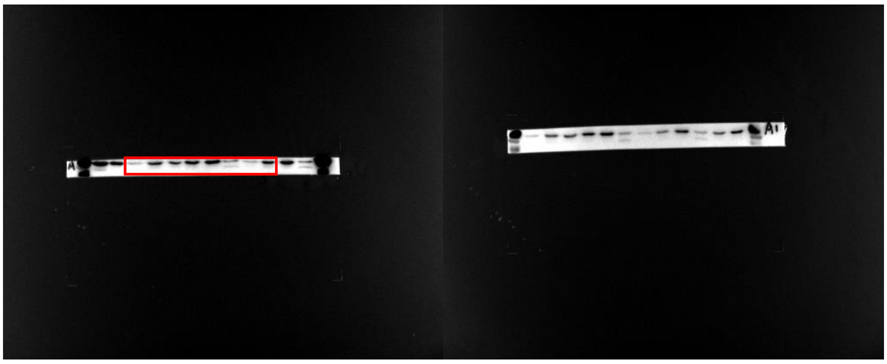

β-actin

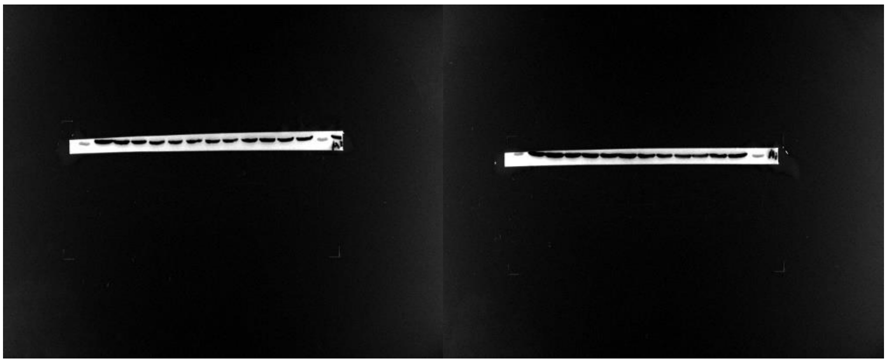

Figure 1P

ALKBH4

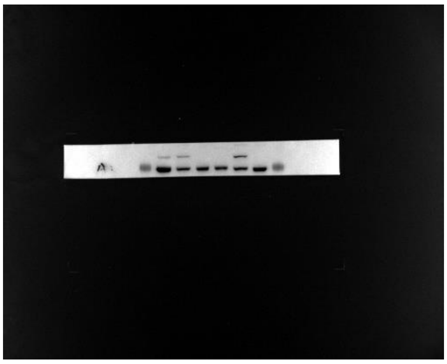

β-actin

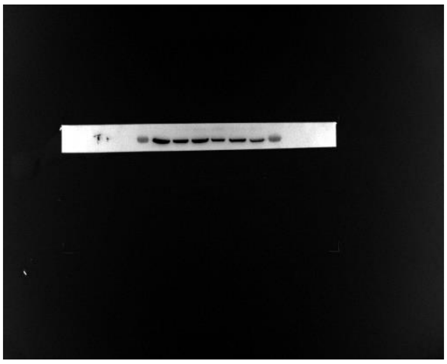

Figure 3C

ALKBH4

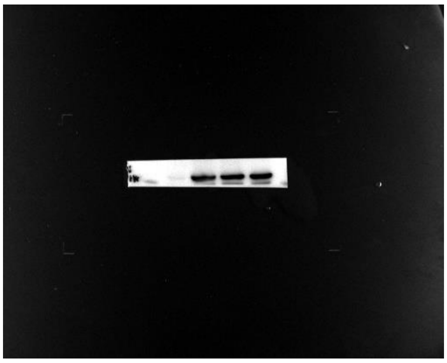

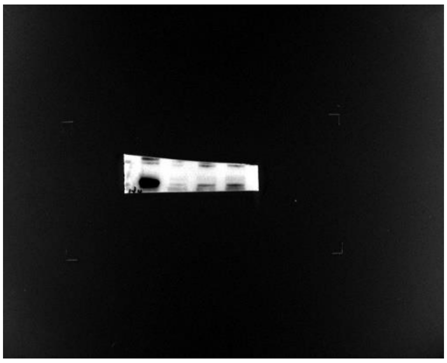

GSDME

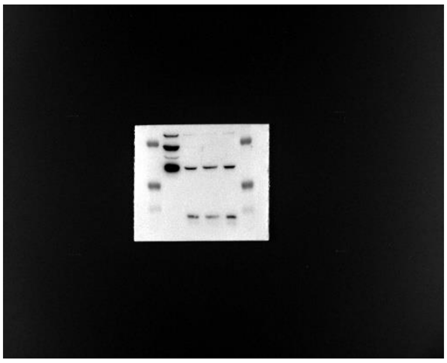

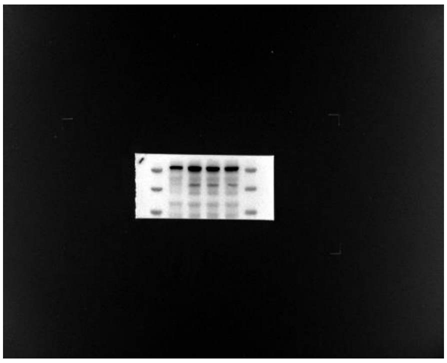

NLRP3

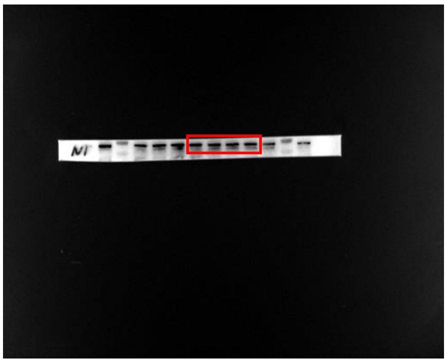

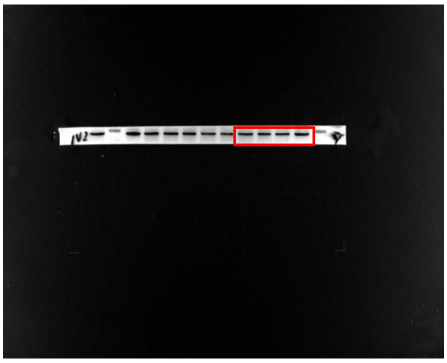

Caspase1

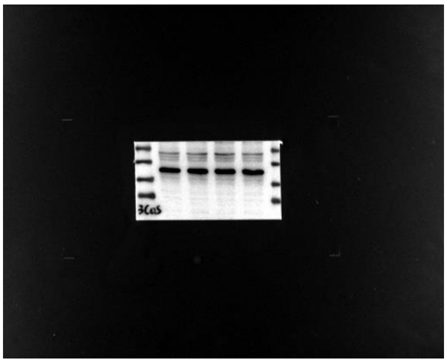

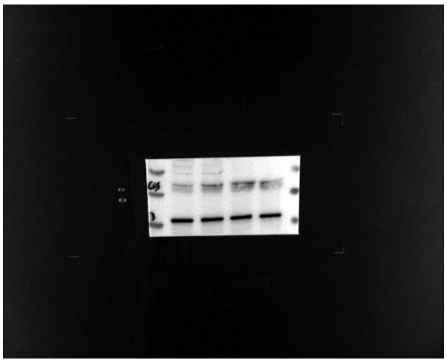

GSDMD

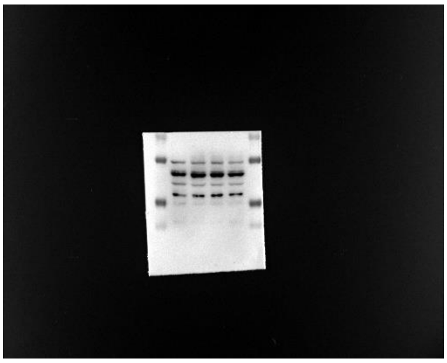

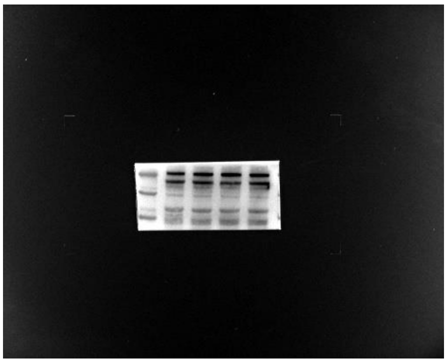

β-actin

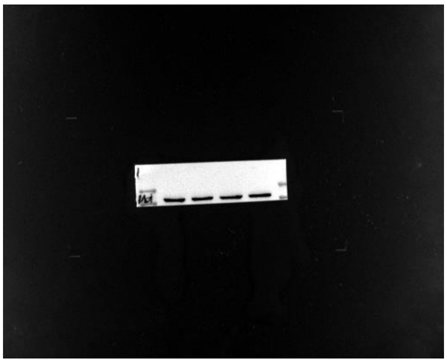

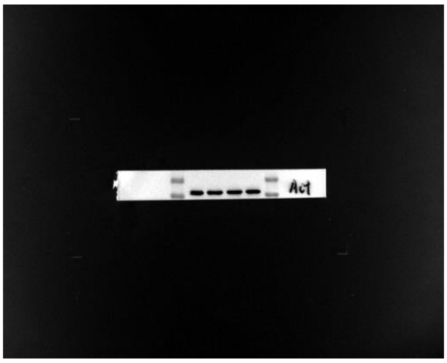

Figure 3D

ALKBH4

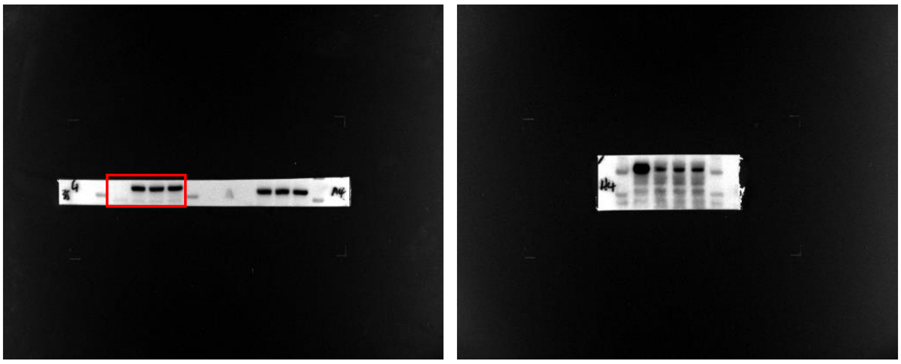

GSDME

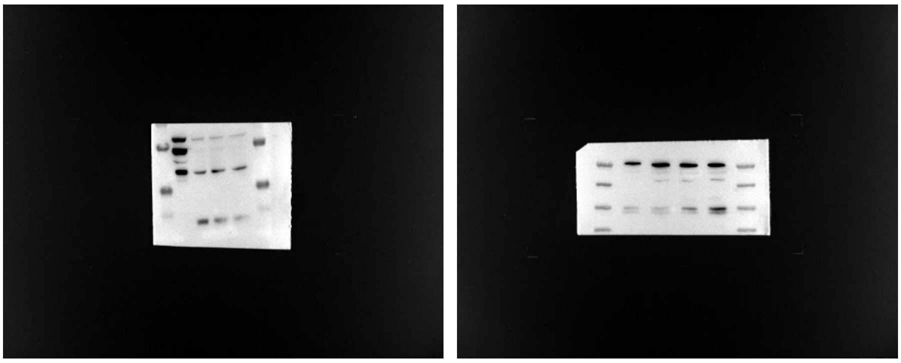

NLRP3

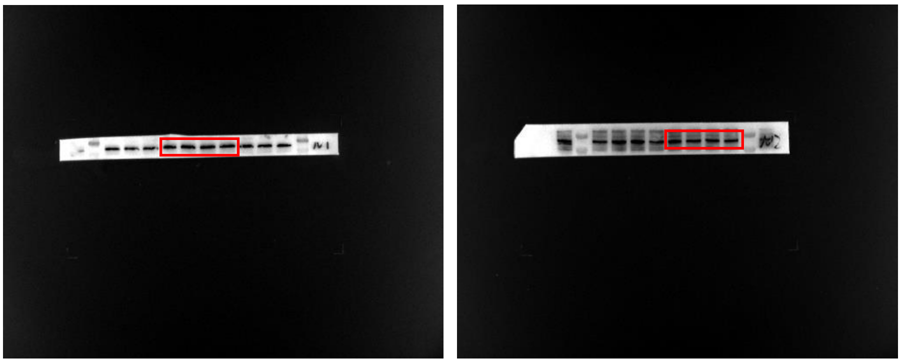

Caspase1

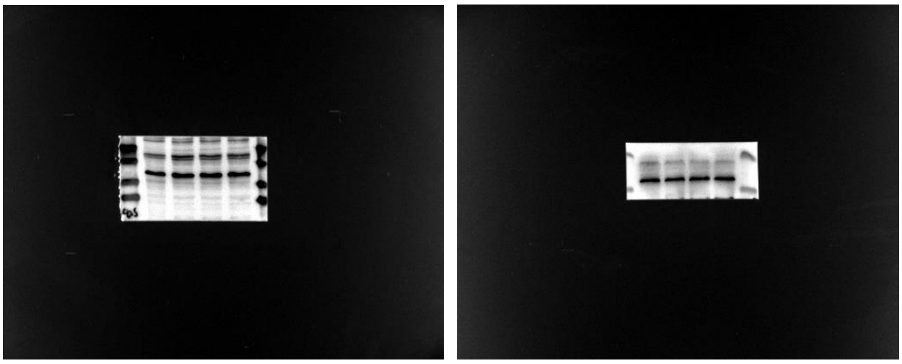

GSDMD

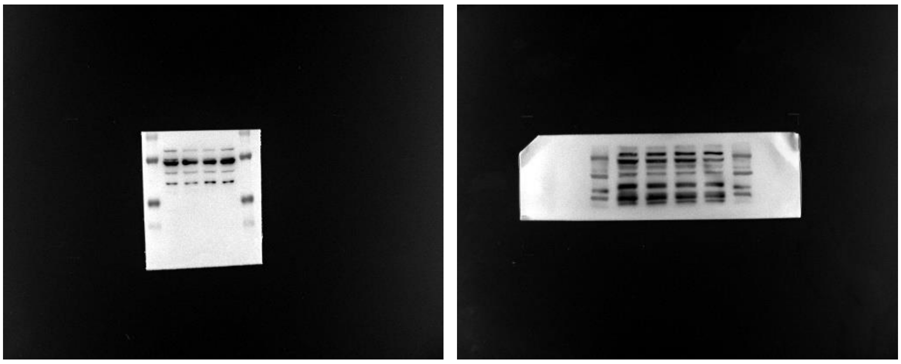

β-actin

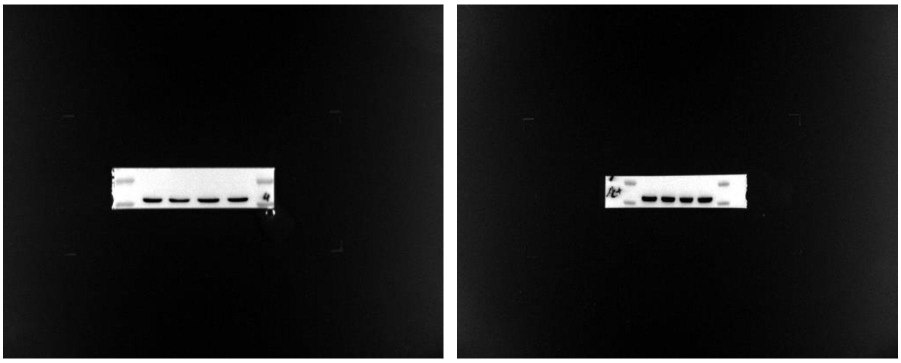

Figure 4D

ALKBH4

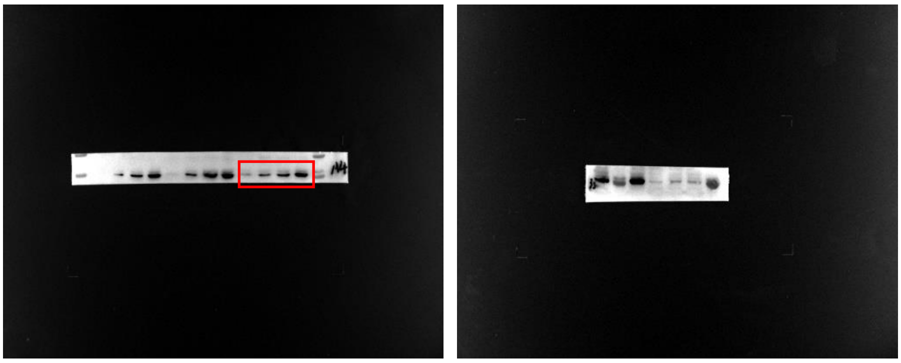

H3K4me3

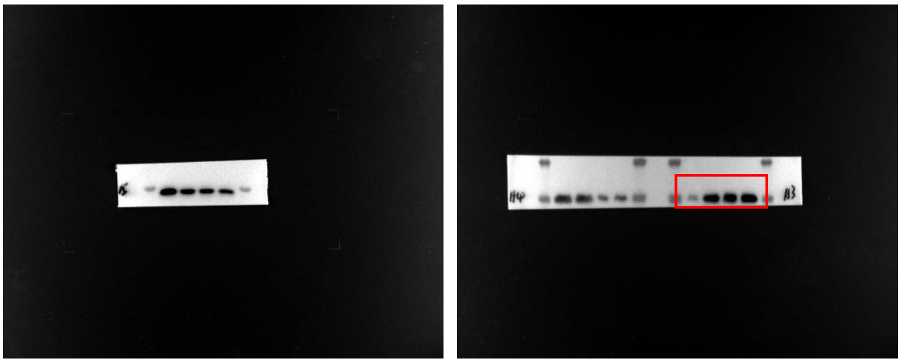

H3K4me1

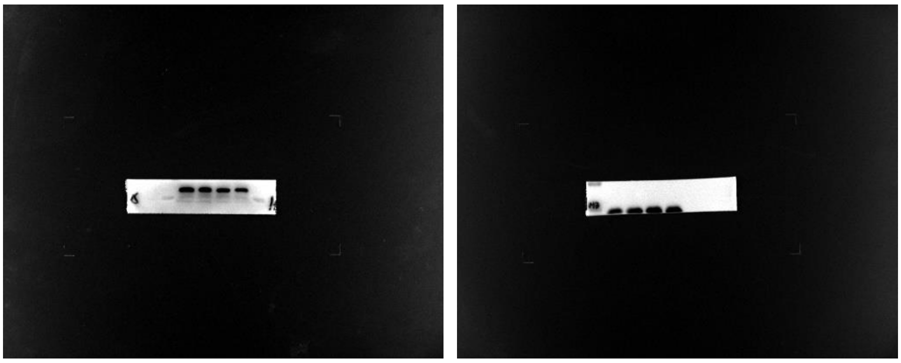

H3K9me3

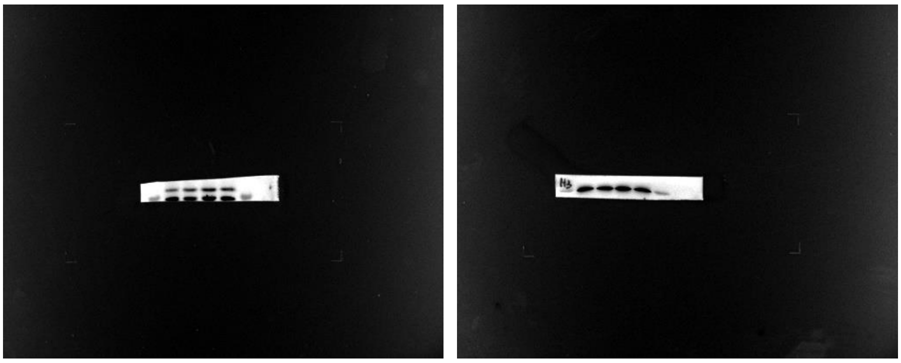

H3K36me3

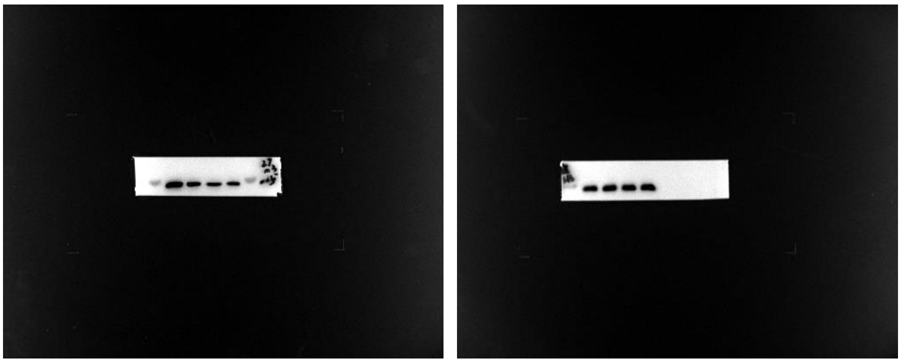

H3K56me3

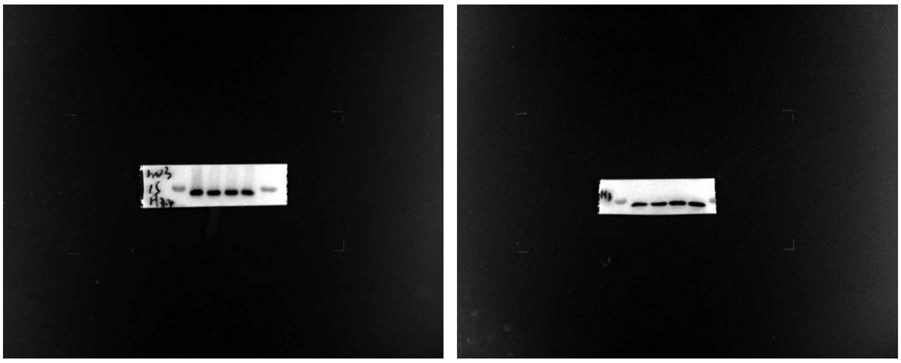

H3K64me3

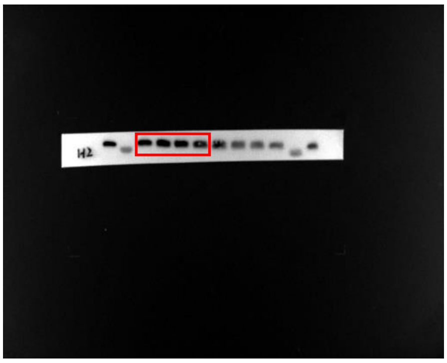

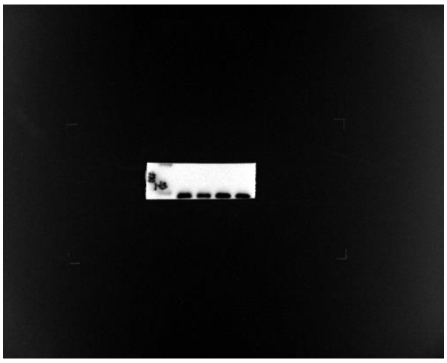

Histone-3

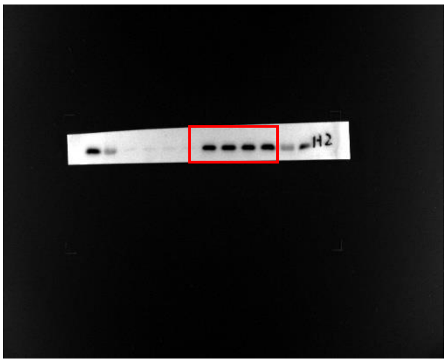

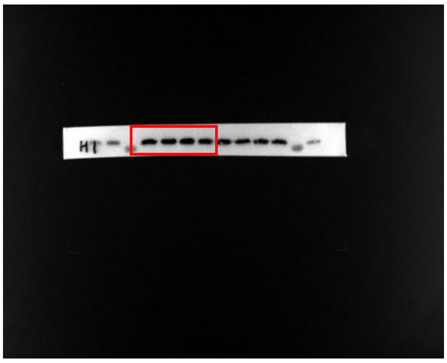

Figure 4E

ALKBH4

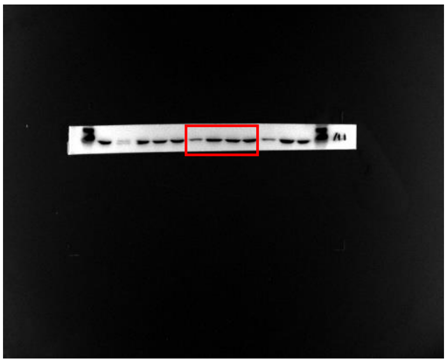

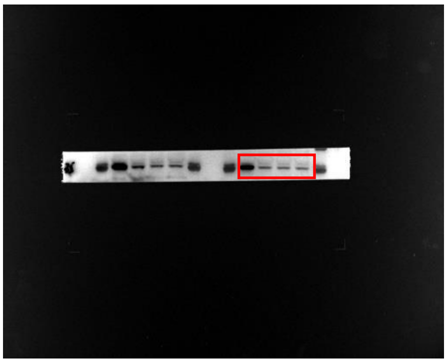

H3K4me3

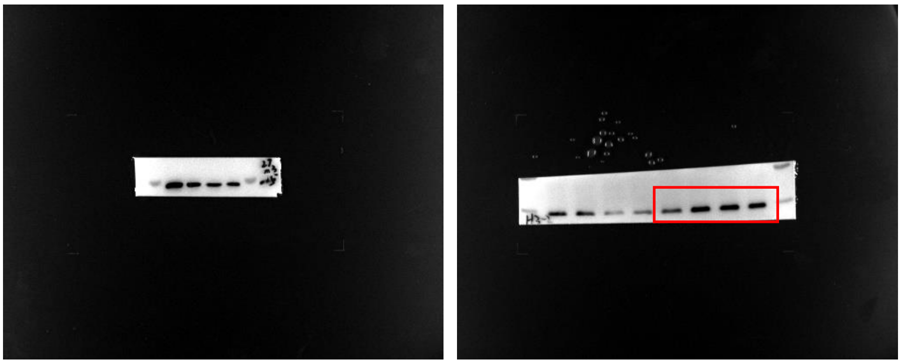

H3K4me1

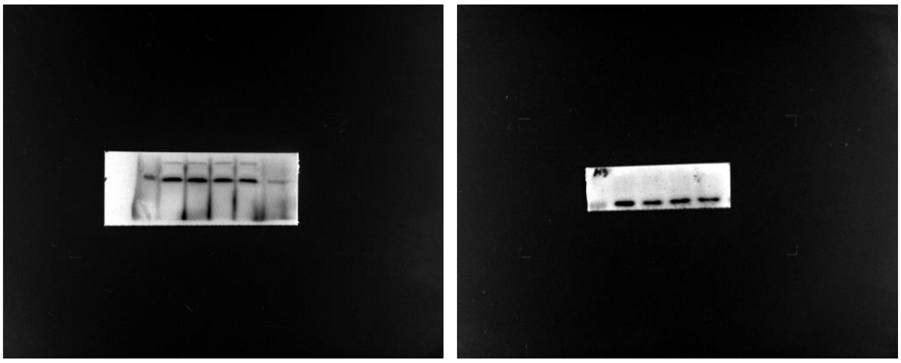

H3K9me3

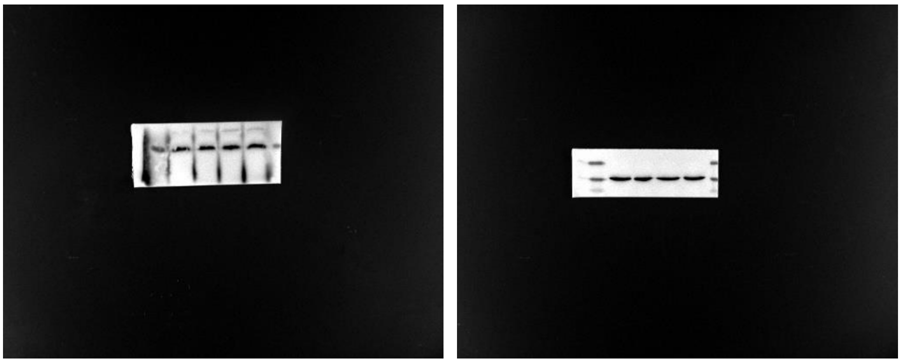

H3K36me3

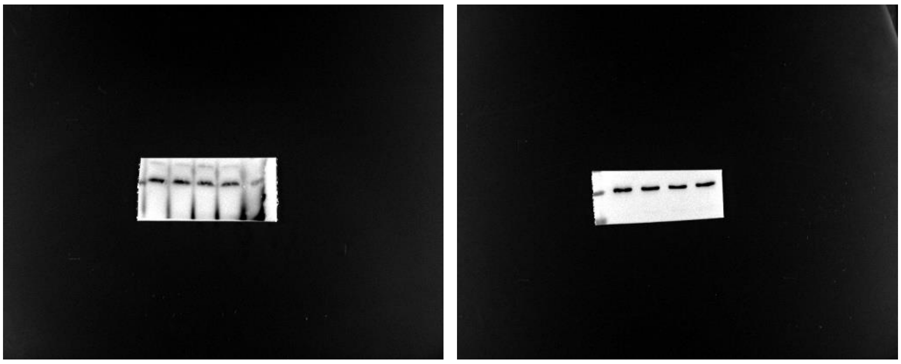

H3K56me3

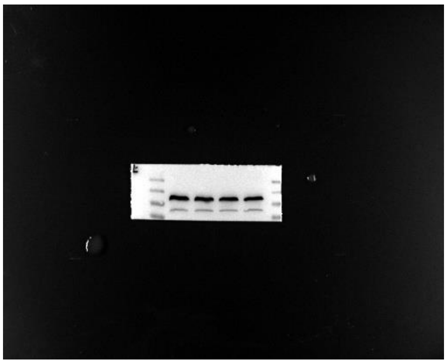

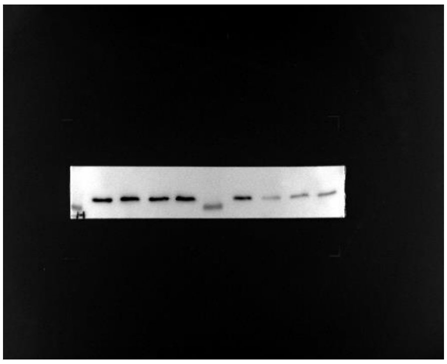

H3K64me3

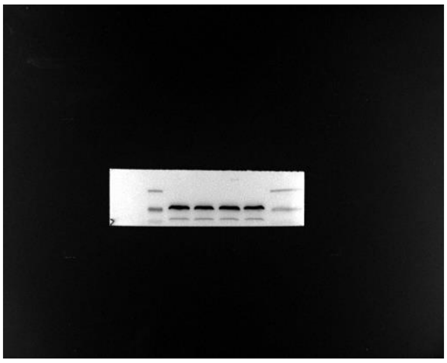

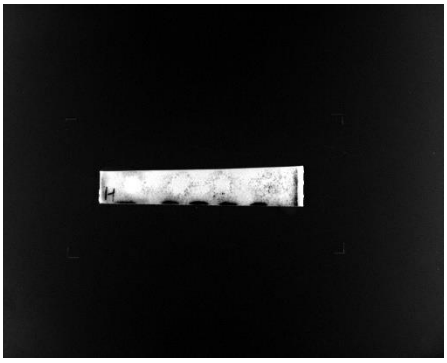

Histone-3

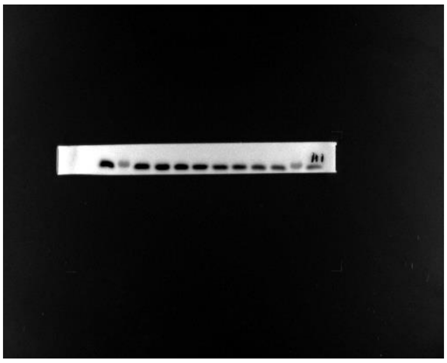

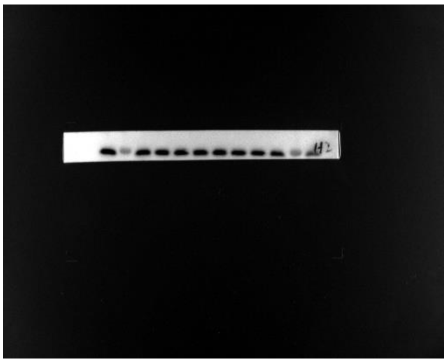

Figure 6H

GSDME

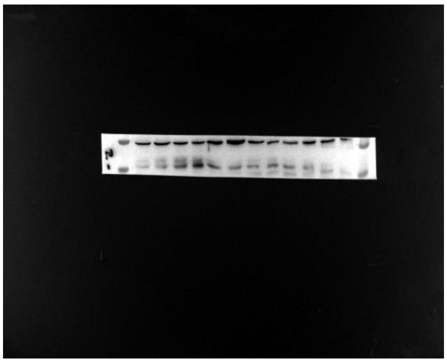

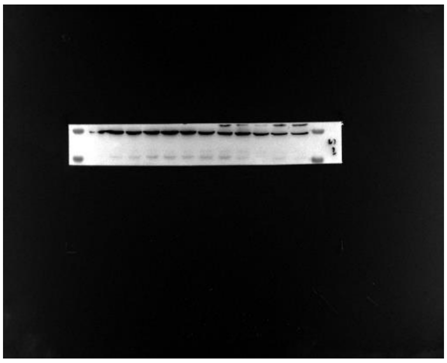

C-Caspase3

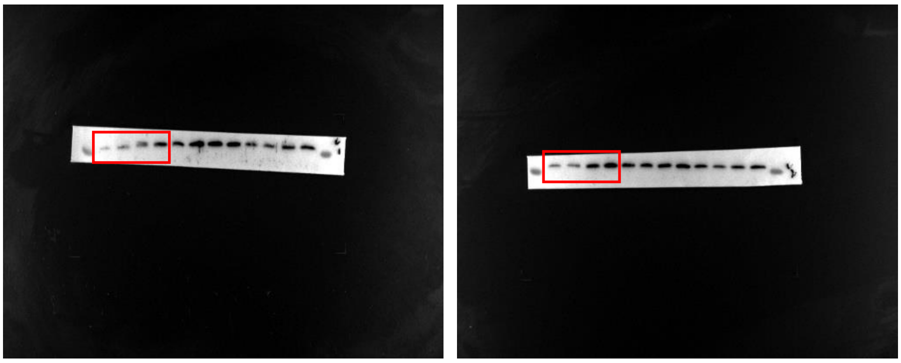

β-actin

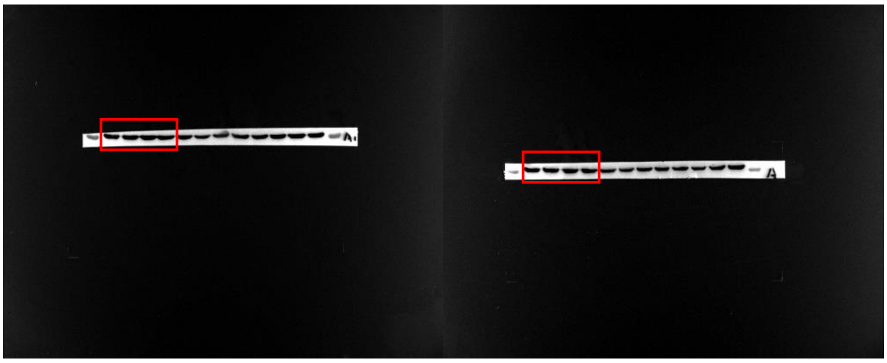

Figure 6I

ALKBH4

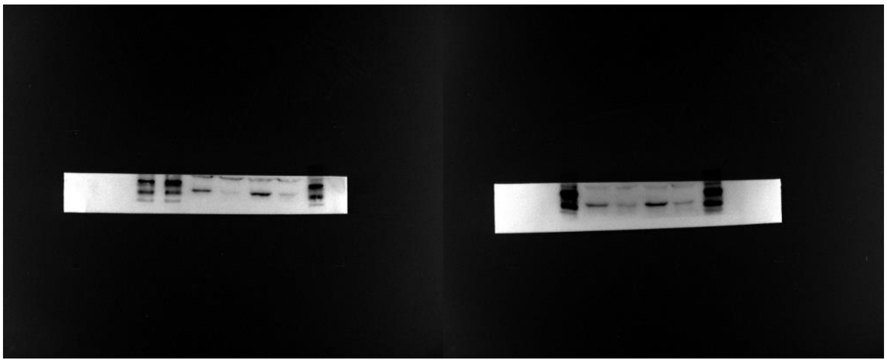

GSDME

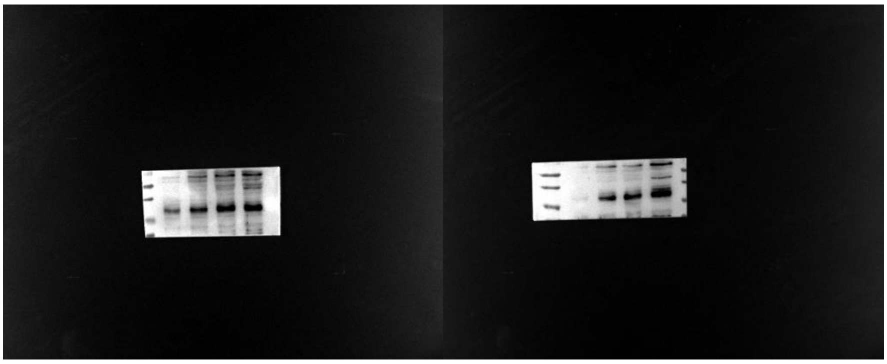

C-Caspase3

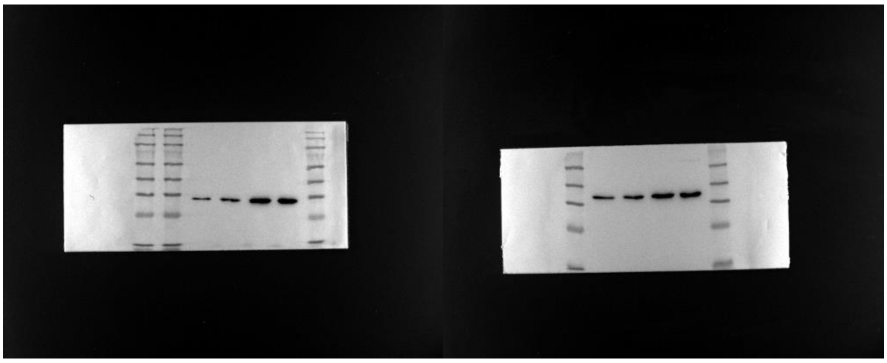

β-actin

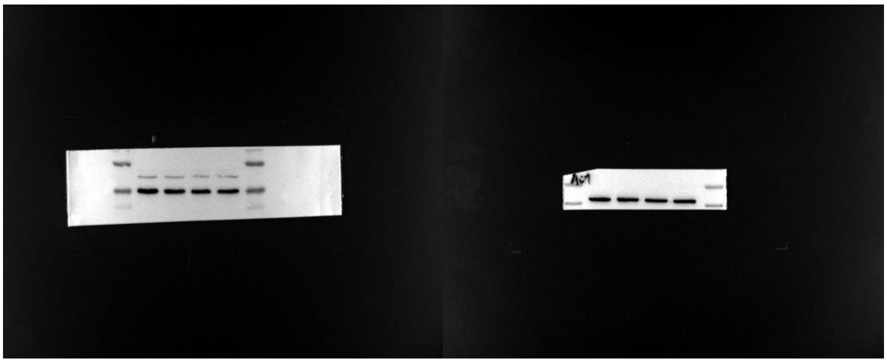

Figure S1C

ALKBH1

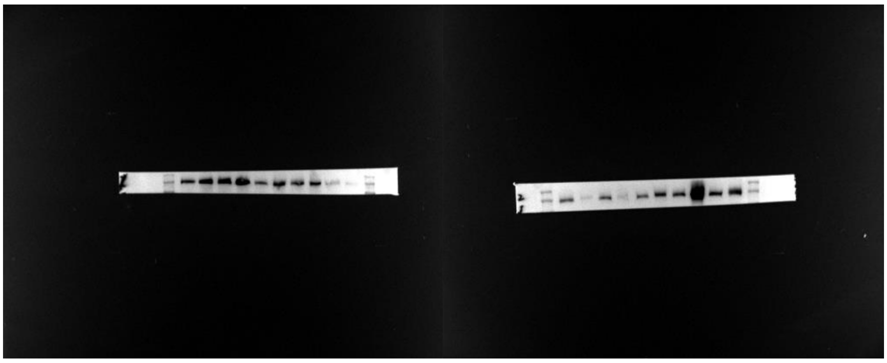

ALKBH2

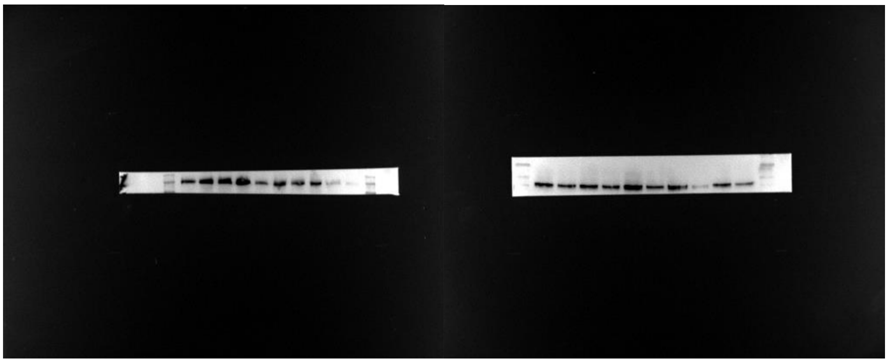

ALKBH3

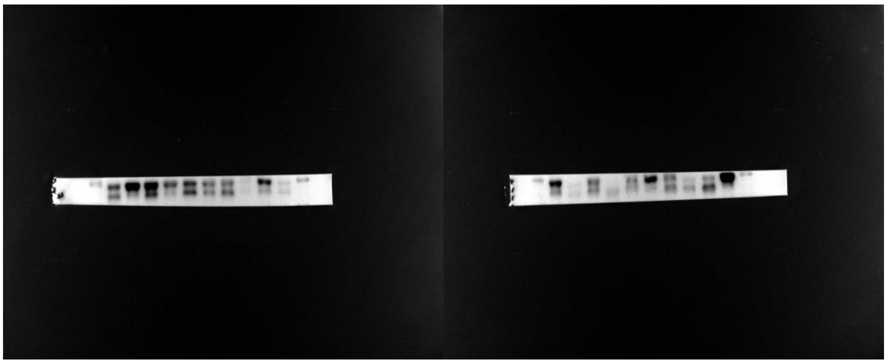

ALKBH5

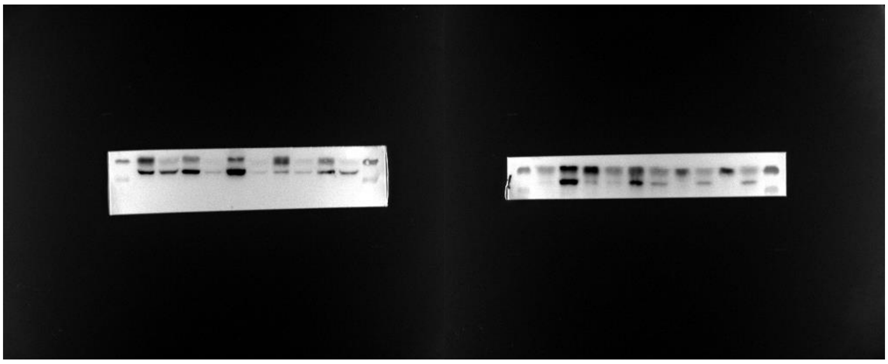

ALKBH6

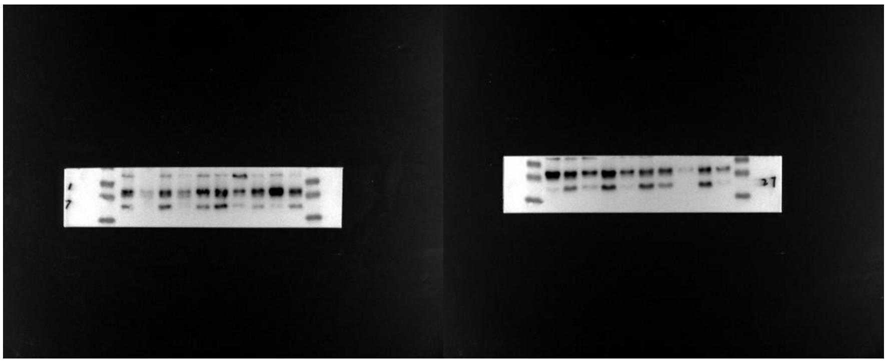

ALKBH7

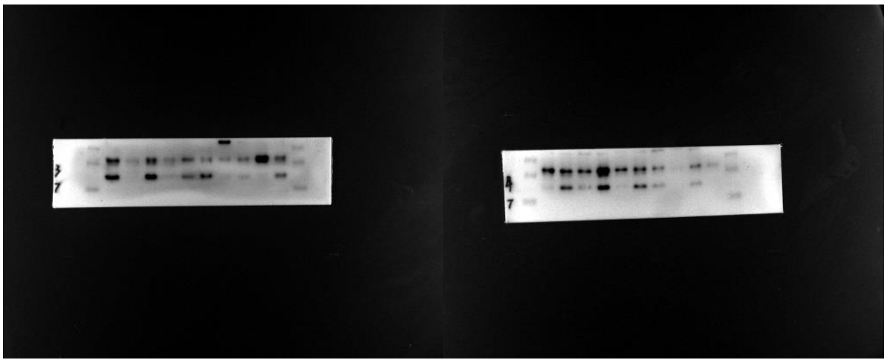

ALKBH8

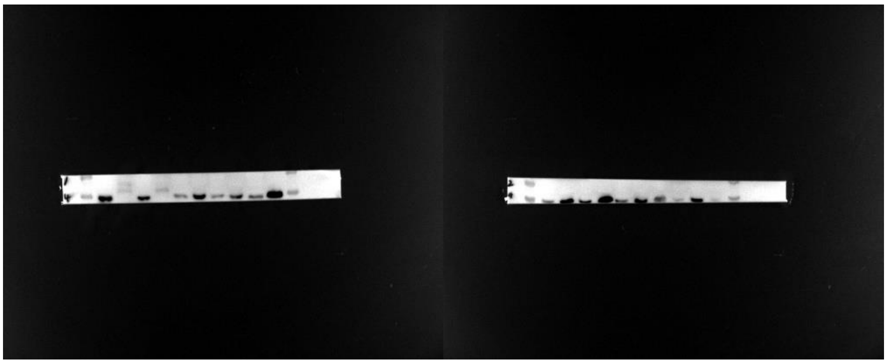

FTO

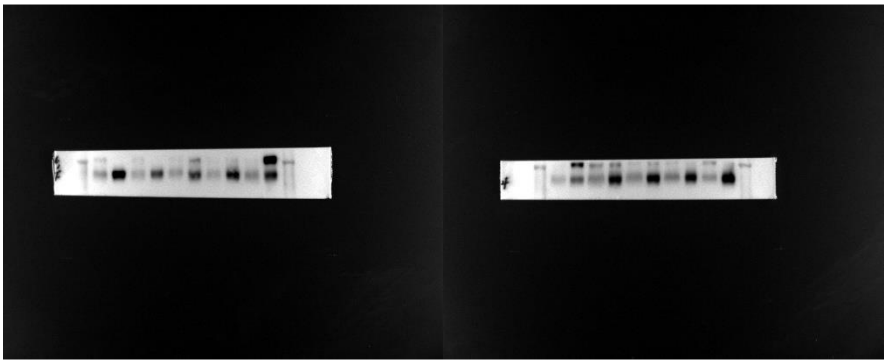

GAPDH

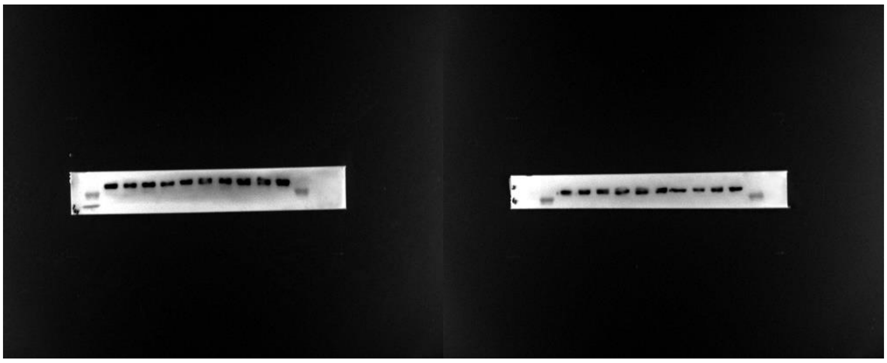

Figure S1D

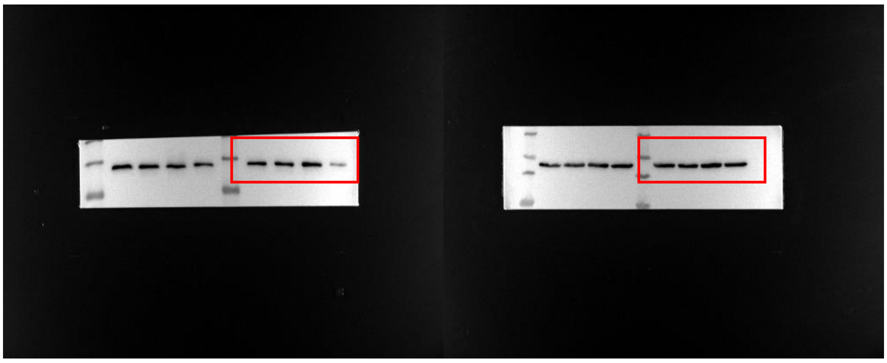

Supplement: Supplementary file 2 — original experimental data [file 41419_2024_6832_MOESM2_ESM.pdf]
